# Frailty for cardiovascular risk stratification in CKM syndrome stages 0–3: a UK Biobank prospective cohort study

**DOI:** 10.3389/fcvm.2026.1918446

**Published:** 2026-08-05

**Authors:** Junhong Wang, Ying Zhao, Zongzong Ma

**Affiliations:** 1 Department of Neurosurgery, The First Affiliated Hospital of Zhengzhou University, Zhengzhou, Henan Province, China; 2 Yunnan Provincial Key Laboratory of Public Health and Biosafety & School of Public Health, Kunming Medical University, Kunming, China; 3 Department of Neurosurgery, Nanshi Hospital of Nanyang, Nanyang, Henan Province, China

**Keywords:** cardiovascular disease, CKM syndrome, frailty index, physical frailty, UK Biobank

## Abstract

**Background:**

Frailty shares common pathophysiological mechanisms with cardiovascular-kidney-metabolic (CKM) syndrome and may contribute to cardiovascular risk. However, evidence regarding the associations of physical frailty and frailty index with cardiovascular outcomes in individuals with CKM stages 0–3 remains limited.

**Methods:**

This prospective cohort study included 330,197 participants with CKM stages 0–3 from the UK Biobank. Physical frailty was assessed using a modified Fried frailty phenotype, while frailty index was constructed based on a 49-item deficit accumulation model. Incident cardiovascular disease (CVD), including coronary heart disease (CHD), stroke, heart failure (HF), atrial fibrillation (AF), and peripheral artery disease (PAD), was identified using ICD-10 codes. Associations were evaluated using cause-specific Cox proportional hazards models after adjustment for demographic, socioeconomic, lifestyle, and clinical factors. Restricted cubic spline, subgroup, and sensitivity analyses were further conducted to assess dose–response relationships and the robustness of the findings.

**Results:**

Both physical frailty and frailty index were significantly associated with increased risks of CVD and all major cardiovascular subtypes (all *p* < 0.001). For overall CVD, each one-score increase in physical frailty was associated with a 21% higher risk (HR: 1.21, 95% CI: 1.20–1.22), while each 0.1-unit increase in frailty index was associated with a 40% higher risk (HR: 1.40, 95% CI: 1.38–1.42). Significant dose-response relationships were observed, and frailty index generally provided greater incremental predictive value than physical frailty.

**Conclusions:**

Among individuals with CKM stages 0–3, both physical frailty and frailty index were independently associated with increased risks of incident CVD and its major subtypes. Frailty assessment may provide complementary information for cardiovascular risk stratification within the CKM continuum.

## Graphical Abstract

**Figure:**
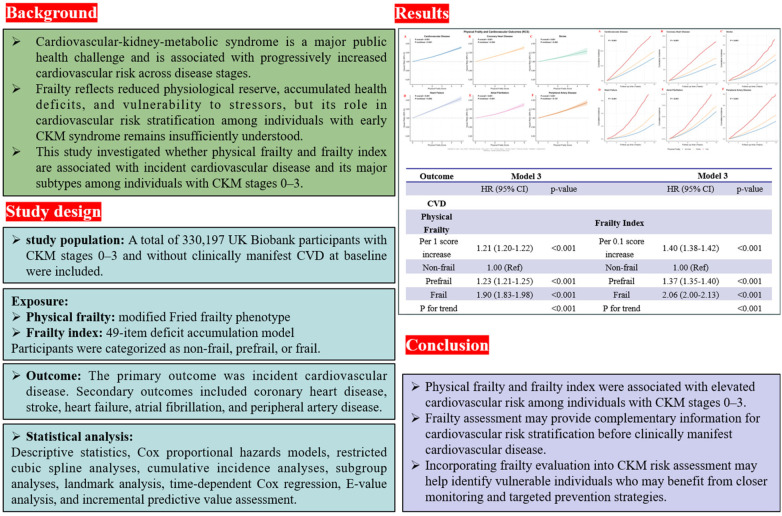

## Introduction

Cardiovascular-kidney-metabolic (CKM) syndrome was formally recognized as a distinct clinical entity by the American Heart Association in 2023 and is characterized by complex bidirectional interactions among cardiovascular dysfunction, kidney disease, and metabolic disorders (1). CKM syndrome has become a major public health challenge, with nearly 90% of U.S. adults exhibiting at least stage 1 CKM syndrome and approximately 15% reaching advanced stages (2). Poor CKM health substantially contributes to premature morbidity and mortality and imposes considerable socioeconomic burdens (2, 3). Consequently, developing comprehensive and life course-oriented prevention strategies to improve CKM health has become an urgent public health priority. Moreover, previous studies have demonstrated a progressive increase in cardiovascular disease (CVD) risk across CKM stages, underscoring the importance of early risk stratification and prevention (4).

Frailty, a multidimensional age-related syndrome characterized by reduced physiological reserve and increased vulnerability to stressors, can be conceptualized using different theoretical frameworks. Physical frailty, represented by the Fried frailty phenotype, focuses primarily on biological and functional manifestations, whereas the frailty index, based on the cumulative deficit model, reflects the multidimensional accumulation of health deficits, including diseases, symptoms, and functional impairments (5–7). Increasing evidence suggests a close relationship between frailty and CKM syndrome, as both conditions share common pathophysiological mechanisms, including chronic inflammation, insulin resistance, oxidative stress, and metabolic dysregulation (8–10). Moreover, frailty has been associated with increased risks of functional decline, hospitalization, and mortality (11, 12).

However, evidence regarding the associations of physical frailty and frailty index with incident cardiovascular outcomes among individuals with CKM stages 0–3 remains limited. Although frailty is often considered a downstream manifestation of ageing and accumulated comorbidity, it may capture reduced physiological reserve and vulnerability that are not fully reflected by conventional cardiometabolic risk factors. To our knowledge, this is the first study within the UK Biobank cohort to simultaneously investigate the associations of both physical frailty and frailty index with incident cardiovascular disease across CKM stages 0–3. Because CKM stages 0–3 represent a continuum of cardiometabolic vulnerability before clinically overt cardiovascular disease, identifying vulnerable individuals during this period may help refine cardiovascular risk stratification and support targeted preventive strategies. Therefore, this study aimed to investigate the associations of physical frailty and frailty index with incident CVD and its major subtypes among individuals with CKM stages 0–3.

## Methods

### Study design and participants

Data for the present study were obtained from the UK Biobank, a large-scale prospective cohort study that recruited more than 500,000 participants from England, Scotland, and Wales between 2006 and 2010. At baseline, participants completed touchscreen-based questionnaires, underwent standardized physical assessments, and provided biological samples. Detailed information regarding the study design, recruitment procedures, and cohort characteristics has been described previously (13). The UK Biobank received ethical approval from the North West Multicentre Research Ethics Committee (reference: 11/NW/0382), and all participants provided written informed consent prior to enrollment. The present study was conducted in accordance with the principles of the Declaration of Helsinki and relevant institutional and national regulations. As this study involved secondary analysis of anonymized UK Biobank data, no additional ethical approval or participant consent was required.

**Figure 1:**
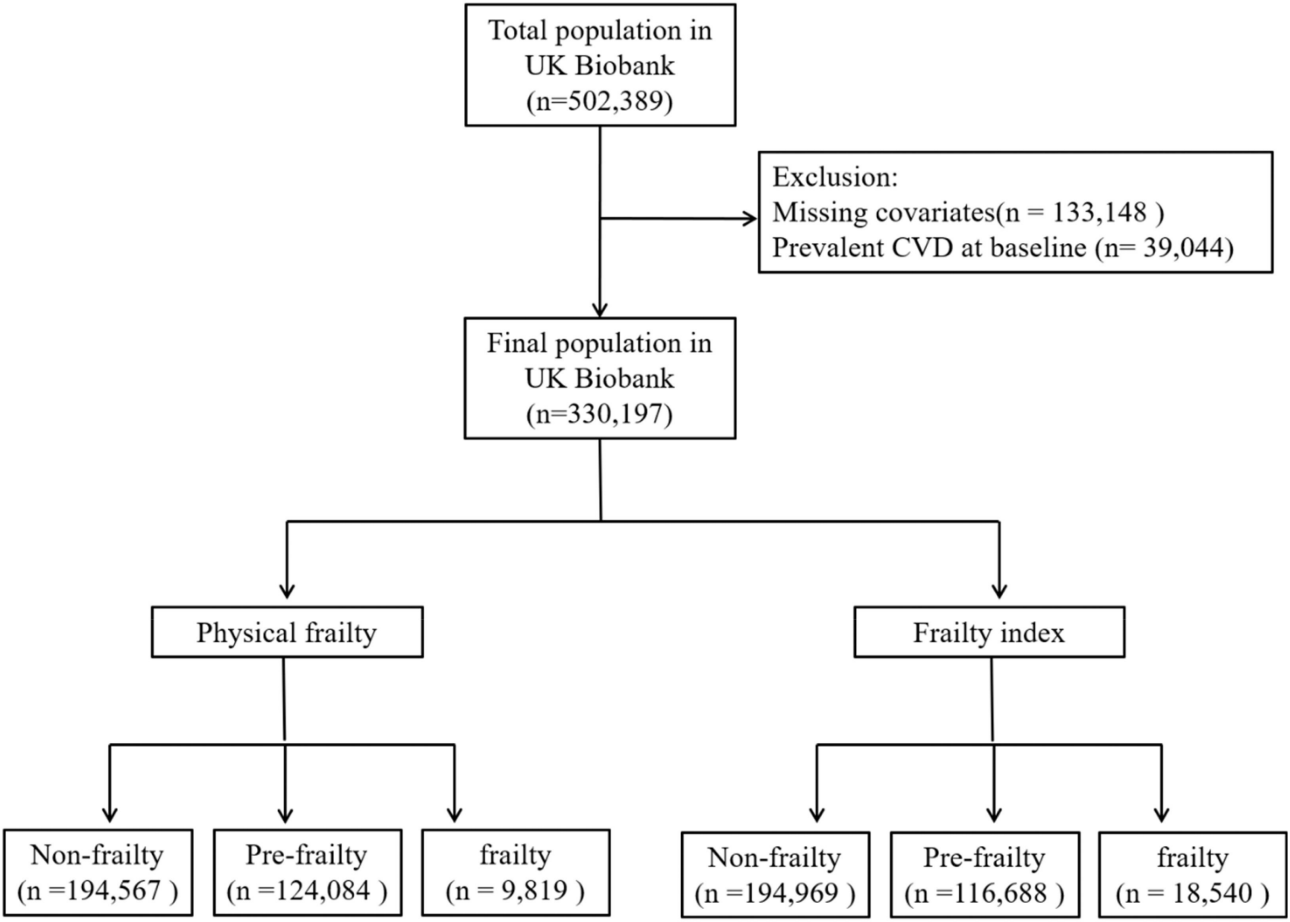
Flowchart of selecting study population in the UK Biobank. CVD: cardiovascular disease.

A total of 502,389 participants were initially enrolled in the UK Biobank cohort. Participants with missing covariate information (*n* = 133,148) were first excluded. To minimize potential reverse causation, individuals with pre-existing cardiovascular disease at baseline, including coronary heart disease (CHD), stroke, heart failure (HF), atrial fibrillation (AF), and peripheral artery disease (PAD), were subsequently excluded (*n* = 39,044). Following the application of these eligibility criteria, 330,197 participants remained and were included in the final analysis. The participant selection process is illustrated in Figure 1, and detailed information regarding missing data across covariates is presented in Supplementary Table S1.

### Definition of CKM syndrome stages 0–3

CKM syndrome stages were classified according to the 2023 American Heart Association Presidential Advisory on CKM Health (3). Briefly, stage 0 included individuals without identifiable CKM-related risk factors. Stage 1 comprised participants with early metabolic abnormalities, including overweight or obesity, abdominal adiposity, and impaired glucose metabolism. Stage 2 was characterized by established metabolic disorders, such as diabetes mellitus, hypertension, hypertriglyceridemia, or metabolic syndrome, as well as moderate-to-high-risk chronic kidney disease (CKD). Stage 3 included individuals with subclinical CVD or very-high-risk CKD. Stage 4 represented overt clinical CVD, including CHD, stroke, HF, AF, and PAD, occurring in combination with features from stages 1–3. Detailed definitions and classification criteria for CKM stages 0–3 are provided in Supplementary Tables S2–S6. Participants classified as stage 4 were excluded from the present analysis because the study focused on incident cardiovascular outcomes in individuals with CKM stages 0–3.

### Exposure assessment

Physical frailty was evaluated using a modified version of the Fried frailty phenotype adapted for the available UK Biobank data (5). Five components were considered, including unintentional weight loss, exhaustion, reduced physical activity, slow walking pace, and low handgrip strength (Supplementary Table S7). Participants were categorized according to the number of fulfilled criteria as non-frail (0 criteria), prefrail (1–2 criteria), or frail (≥3 criteria) (5, 14).

Frailty index was constructed according to the cumulative deficit framework proposed by Rockwood and colleagues, in which frailty is quantified as the proportion of accumulated health deficits across multiple domains (15). The selection and scoring of deficits were based on available UK Biobank variables and were informed by previously published deficit-accumulation frailty indices, including the electronic frailty index and the UK Biobank-based frailty index (6, 16, 17). The final 49-item frailty index covered multiple domains, including sensory impairment, mental wellbeing, infirmity, cardiometabolic conditions, respiratory and musculoskeletal disorders, cancer, pain, and gastrointestinal conditions. For participants with fewer than 10 missing items, the frailty index score was calculated as the proportion of deficits present relative to the total number of assessed deficits, yielding a continuous score ranging from 0 to 1. Based on established cut-off values, participants were classified as non-frail (≤0.12), prefrail (>0.12–0.24), or frail (>0.24). This approach differs from the phenotype-based frailty measure by capturing multidimensional health deficits rather than physical manifestations alone.

### Ascertainment of cardiovascular outcomes

Incident cardiovascular disease (CVD) was defined as the primary outcome among participants with CKM syndrome stages 0–3. The composite CVD outcome comprised five major cardiovascular conditions: coronary heart disease (CHD), stroke, heart failure (HF), atrial fibrillation (AF), and peripheral artery disease (PAD). Disease outcomes were identified using the International Classification of Diseases, Tenth Revision (ICD-10) codes. Specifically, CHD was defined by codes I20, I21, I22, I24, and I25; stroke by codes I60–I64; HF by code I50; AF by code I48; and PAD by codes I70–I74 and I77–I79 (Supplementary Table S3). For each outcome, the first documented event during follow-up was considered the endpoint of interest. Follow-up time was calculated from the date of baseline assessment to the first occurrence of the outcome of interest, death, or the end of follow-up, whichever occurred first.

### Covariates

Baseline demographic characteristics, including age, sex, ethnicity, and educational attainment, were collected through touchscreen-based questionnaires administered at the assessment centers. Socioeconomic status was assessed using the Townsend deprivation index, which was derived from participants' residential postal codes linked to national census data. Lifestyle-related variables included smoking status (never, former, or current), alcohol consumption frequency (never, <3 times/week, or ≥3 times/week), sleep duration (<6, 6–9, or >9 h/day), and physical activity level (low, moderate, or high). Clinical characteristics comprised body mass index (BMI), waist circumference, hypertension, diabetes mellitus, and dyslipidemia. Hypertension and diabetes were identified based on self-reported physician diagnoses, measured clinical parameters, or the use of corresponding medications. Laboratory measurements included triglycerides, fasting plasma glucose, creatinine, cystatin C, hemoglobin, high-density lipoprotein cholesterol (HDL-C), and total cholesterol.

### Statistical analysis

Normally distributed continuous variables are presented as mean ± standard deviation (SD), whereas skewed continuous variables are expressed as median with interquartile range (IQR). Categorical variables are summarized as frequencies and percentages. Incidence rates were calculated as the number of events per 1,000 person-years. Cumulative incidence curves for CVD outcomes were generated according to physical frailty status and frailty index categories and compared using Gray's test. Associations of physical frailty and frailty index with incident CVD outcomes were evaluated using cause-specific Cox proportional hazards models, with results reported as hazard ratios (HRs) and corresponding 95% confidence intervals (CIs).

Three progressively adjusted models were constructed. Model 1 included age, sex, and ethnicity. Model 2 was additionally adjusted for educational attainment, BMI, Townsend deprivation index, smoking status, alcohol consumption frequency, physical activity level. Model 3 further adjusted for hypertension, diabetes mellitus. Variables included in multivariable models were selected based on clinical relevance and potential collinearity with frailty-related components. Both physical frailty and frailty index were analyzed as continuous and categorical variables. For continuous analyses, association for physical frailty was evaluated per 1-score increase, whereas frailty index was evaluated per 0.1-unit increase. For categorical analyses, participants were classified as non-frail, prefrail, and frail according to predefined criteria, with the non-frail group serving as the reference category.

To further evaluate whether the associations between frailty and incident CVD differed according to CKM severity, additional analyses were performed stratified by CKM stages 0, 1, 2, and 3. In these analyses, physical frailty and frailty index were separately examined within each CKM stage. For the primary overall analyses, Model 3 further adjusted for hypertension and diabetes mellitus. However, for the CKM stage-stratified analyses, hypertension and diabetes mellitus were not included in the primary adjustment model because these conditions are components of CKM staging and may overlap with frailty index construction, potentially resulting in over-adjustment.

Restricted cubic spline (RCS) models were used to examine the dose-response relationships of the physical frailty score and frailty index with incident cardiovascular disease. Candidate spline models with different knot specifications were compared using the Akaike information criterion, and the model with the lowest Akaike information criterion was selected. Accordingly, three knots were used for each frailty measure. For the physical frailty score, a discrete variable ranging from 0 to 5, the knots were placed at scores of 1, 2, and 4. For the frailty index, the knots were placed at the 10th, 50th, and 90th percentiles, corresponding to values of 0.0663, 0.1607, and 0.2806, respectively. Tests for overall association and nonlinearity were performed using likelihood ratio tests.

Subgroup analyses were performed according to age, sex, smoking status, alcohol consumption, diabetes mellitus and hypertension. Potential effect modification was evaluated using likelihood ratio tests by comparing models with and without corresponding interaction terms. Associations of physical frailty and frailty index with incident CVD outcomes were examined within each subgroup using the fully adjusted Cox proportional hazards models. Physical frailty and frailty index were analyzed according to predefined categories (non-frail, prefrail, and frail), with the non-frail group serving as the reference category.

Several sensitivity analyses were conducted to examine the robustness of the primary findings. First, time-dependent Cox regression analyses were performed to assess whether the associations of physical frailty and frailty index with incident CVD outcomes varied across different follow-up periods (0–5, 5–10, and >10 years). Second, a 2-year landmark analysis was conducted by excluding participants who developed CVD events within the first two years of follow-up to reduce the potential influence of reverse causation. Third, E-value analyses were performed to quantify the minimum magnitude of unmeasured confounding required to explain away the observed associations. Fourth, to address potential overlap between frailty index components and CKM staging criteria, we additionally constructed a modified 42-item frailty index for sensitivity analysis. Seven cardiometabolic and cardiovascular-related deficits were excluded from the original 49-item frailty index, including diabetes, heart attack, angina, stroke, hypertension, deep vein thrombosis, and cholesterol-lowering medication use. The remaining 42 deficits were combined using the same cumulative deficit approach. The modified frailty index was calculated as the proportion of deficits present among the available items, and participants with fewer than 35 available items were excluded from this sensitivity analysis. The same cut-off values were used to define non-frail, prefrail, and frail categories. Fifth, to assess the potential influence of missing data, we performed a sensitivity analysis using multiple imputation by chained equations. Twenty imputed datasets were generated. CKM stage was not imputed directly; instead, its component variables were imputed, and CKM stage was recalculated within each completed dataset. Cox proportional hazards models were fitted separately in each imputed dataset, and the regression coefficients and standard errors were combined according to Rubin's rules. The resulting estimates were compared with those obtained from the complete-case analysis. Sixth, to account for the possibility that death could preclude the occurrence or ascertainment of incident CVD, we additionally performed competing-risk analyses using Fine–Gray subdistribution proportional hazards models, with death occurring before incident CVD treated as the competing event. Subdistribution hazard ratios (sHRs) and 95% confidence intervals (CIs) were estimated using the same exposure definitions and covariate adjustment sets as those used in the primary Cox proportional hazards models. The cause-specific Cox models were retained as the primary analyses, whereas the Fine–Gray models were used as complementary analyses to evaluate the associations of frailty with the cumulative incidence of CVD in the presence of competing mortality.

All statistical analyses were conducted using R software (version 4.3.1). All statistical tests were two-sided, and a *P* value <0.05 was considered statistically significant.

## Results

### Baseline characteristics of the study population

A total of 330,197 participants were included in the final analysis. According to physical frailty status, 194,567 (58.9%), 124,084 (37.6%), and 9,819 (3.0%) participants were classified as non-frail, prefrail, and frail, respectively, while 194,969 (59.0%), 116,688 (35.3%), and 18,540 (5.6%) participants were categorized as non-frail, prefrail, and frail according to the frailty index. Baseline characteristics stratified by frailty index, physical frailty, and CKM stages are presented in Table 1 and Supplementary Tables S9,S10.

**Table 1:** Baseline characteristics of the participants in accordance with frailty index.

Characteristic	Frailty index				*p* value
	Overall	Non-frail	Prefrail	Frail	
*N*	3,30,197	1,94,969	1,16,688	18,540	
Demographic					
Age, years, mean (SD)	56.08 (8.09)	55.67 (8.12)	56.63 (8.04)	56.85 (7.81)	<0.001
Gender, female, (%)	1,48,030 (44.8)	94,515 (48.5)	47,463 (40.7)	6,052 (32.6)	<0.001
Ethnicity, white, *n* (%)	3,11,416 (94.3)	1,84,583 (94.7)	1,09,710 (94.0)	17,123 (92.4)	<0.001
Education level, *n* (%)					<0.001
Low	90,250 (27.3)	46,165 (23.7)	36,486 (31.3)	7,599 (41.0)	
Intermediate	1,30,274 (39.5)	76,817 (39.4)	46,383 (39.7)	7,074 (38.2)	
High	1,09,673 (33.2)	71,987 (36.9)	33,819 (29.0)	3,867 (20.9)	
Townsend deprivation index [mean (SD)]	−1.39 (3.02)	−1.65 (2.88)	−1.15 (3.12)	−0.09 (3.46)	<0.001
Lifestyle factor					
Smoking status, *n* (%)					<0.001
Never	1,83,842 (55.9)	1,15,134 (59.3)	60,225 (51.8)	8,483 (46.0)	
Former	1,11,087 (33.8)	62,174 (32.0)	42,160 (36.3)	6,753 (36.6)	
Current	34,034 (10.3)	17,000 (8.7)	13,825 (11.9)	3,209 (17.4)	
Alcohol consumption, *n* (%)					<0.001
Never	24,712 (7.5)	11,688 (6.0)	10,127 (8.7)	2,897 (15.6)	
< 3 times/week	1,59,462 (48.3)	90,278 (46.3)	58,871 (50.5)	10,313 (55.6)	
≥ 3 times/week	1,46,023 (44.2)	93,003 (47.7)	47,690 (40.9)	5,330 (28.7)	
Sleep duration, *n* (%)					<0.001
<6 h	18,848 (5.7)	6,689 (3.4)	9,015 (7.7)	3,144 (17.0)	
6–9 h	3,06,099 (92.7)	1,86,356 (95.6)	1,05,278 (90.2)	14,465 (78.0)	
>9 h	5,250 (1.6)	1,924 (1.0)	2,395 (2.1)	931 (5.0)	
Physical activity, *n* (%)					<0.001
Low	1,11,406 (33.7)	59,111 (30.3)	43,519 (37.3)	8,776 (47.3)	
Moderate	1,09,386 (33.1)	69,813 (35.8)	35,162 (30.1)	4,411 (23.8)	
High	1,09,405 (33.1)	66,045 (33.9)	38,007 (32.6)	5,353 (28.9)	
Clinical characteristic					
Body mass index, kg/m^2^, [mean (SD)]	27.23 (4.66)	26.57 (4.16)	27.88 (4.94)	29.96 (6.05)	<0.001
Systolic blood pressure, mmHg, [mean (SD)]	137.74 (18.56)	137.49 (18.61)	138.13 (18.55)	137.89 (18.03)	<0.001
Diastolic blood pressure, mmHg, [mean (SD)]	82.42 (10.06)	82.19 (10.06)	82.67 (10.04)	83.14 (10.12)	<0.001
Diabetes, *n* (%)	34,830 (10.5)	13,069 (6.7)	16,912 (14.5)	4,849 (26.2)	<0.001
Hypertension, *n* (%)	2,51,413 (76.1)	1,43,694 (73.7)	92,065 (78.9)	15,654 (84.4)	<0.001
Dyslipidemia, *n* (%)	1,84,140 (55.8)	99,243 (50.9)	71,451 (61.2)	13,446 (72.5)	<0.001
Incident cardiovascular disease, *n* (%)	52,454 (15.9)	25,192 (12.9)	22,006 (18.9)	5,256 (28.3)	<0.001
Incident coronary heart disease, *n* (%)	26,289 (8.0)	11,869 (6.1)	11,362 (9.7)	3,058 (16.5)	<0.001
Incident stroke, *n* (%)	7,604 (2.3)	3,910 (2.0)	3,037 (2.6)	657 (3.5)	<0.001
Incident atrial fibrillation, *n* (%)	19,610 (5.9)	9,766 (5.0)	8,114 (7.0)	1,730 (9.3)	<0.001
Incident heart failure, *n* (%)	8,790 (2.7)	3,709 (1.9)	3,927 (3.4)	1,154 (6.2)	<0.001
Incident peripheral artery disease, *n* (%)	10,975 (3.3)	4,936 (2.5)	4,801 (4.1)	1,238 (6.7)	<0.001
Laboratory parameter					
Triglycerides, mgdl, median (IQR)	130.38 [91.85, 188.83]	124.71 [88.22, 180.86]	136.66 [96.63, 196.71]	152.78 [107.08, 220.45]	<0.001
Glucose, mgdL, median (IQR)	88.65 [82.80, 95.38]	88.42 [82.67, 94.79]	88.92 [82.94, 96.01]	89.86 [83.30, 98.82]	<0.001
Creatinine, mgdL, median (IQR)	0.79 [0.69, 0.91]	0.80 [0.70, 0.92]	0.78 [0.68, 0.90]	0.76 [0.67, 0.88]	<0.001
HDL-C, mg/dL, [mean (SD)]	56.61 (14.75)	57.40 (14.81)	55.80 (14.62)	53.45 (14.28)	<0.001
Total Cholesterol mgdl, [mean (SD)]	223.12 (42.93)	224.46 (41.88)	221.62 (43.99)	218.45 (46.36)	<0.001

HDL-C, high-density lipoprotein cholesterol; SD, standard deviation; IQR, interquartile range.

**Figure 2:**
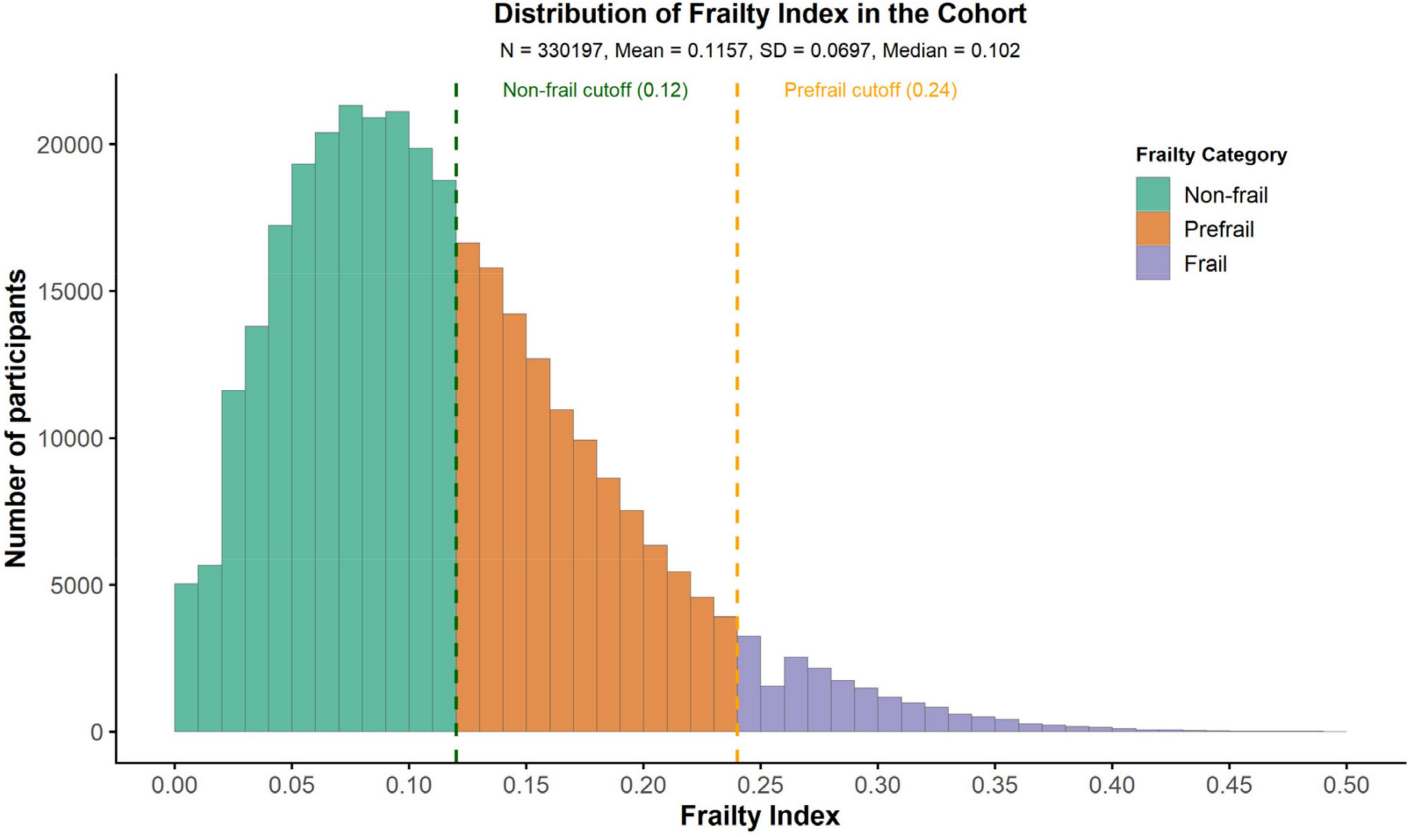
Distribution of frailty index in the cohort.

Across both frailty assessment approaches, increasing frailty severity was associated with older age, greater socioeconomic deprivation, and a higher burden of cardiometabolic risk factors, including hypertension, diabetes, and dyslipidemia (all *p* < 0.001). In addition, the cumulative incidence of overall CVD and its major subtypes progressively increased across frailty categories. Similarly, advancing CKM stages were associated with less favorable demographic, lifestyle, and clinical profiles, accompanied by an increased prevalence of frailty and cardiovascular outcomes (all *p* < 0.001). The distribution of frailty index scores in the study population demonstrated a right-skewed pattern, with the majority of participants classified as non-frail or prefrail and relatively few individuals categorized as frail (Figure 2).

### Associations of physical frailty and frailty index with incident cardiovascular outcomes

**Figure 3:**
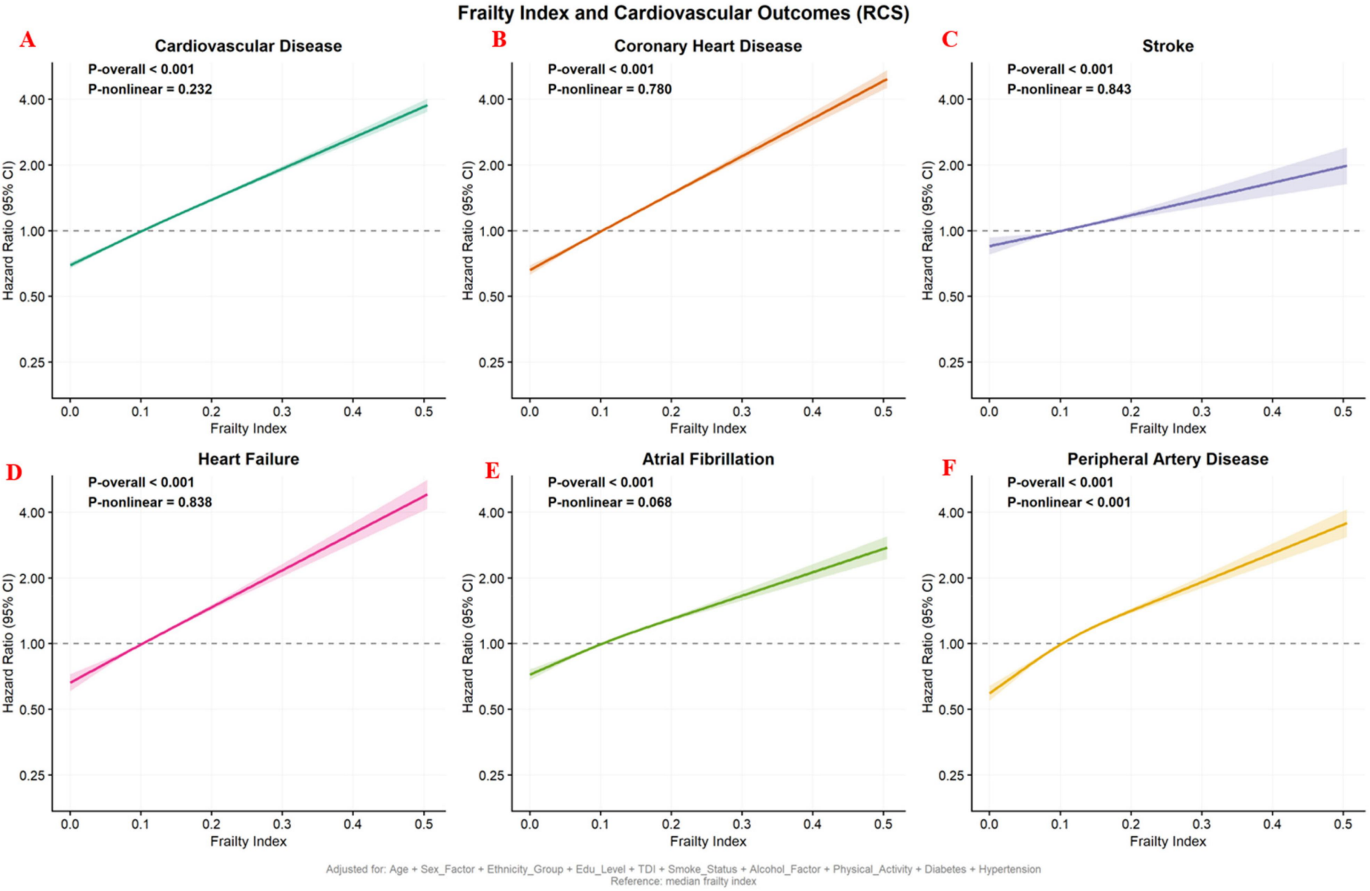
Dose-response associations of frailty index with CVD outcomes. Restricted cubic spline models were fitted using three knots. For the physical frailty score, the knots were placed at scores of 1, 2, and 4. For the frailty index, the knots were placed at the 10th, 50th, and 90th percentiles, corresponding to values of 0.0663, 0.1607, and 0.2806, respectively. Solid lines represent hazard ratios, and shaded areas represent 95% confidence intervals. Adjusted for variables in Model 3. **(A)** Cardiovascular disease; **(B)** coronary heart disease; **(C)** stroke; **(D)** heart failure; **(E)** atrial fibrillation; **(F)** peripheral artery disease.

Associations of physical frailty and frailty index with incident cardiovascular outcomes are presented in Table 2, while dose–response relationships are illustrated in Figure 3 and Supplementary Figure S1. In fully adjusted models (Model 3), both physical frailty and frailty index were significantly associated with an increased risk of CVD and all major cardiovascular subtypes, including CHD, stroke, HF, AF, and PAD (all *p* < 0.001). A graded increase in risk was observed across frailty categories, with significant dose-dependent trends for all outcomes (*P* for trend <0.001).

**Table 2:** Associations of frailty status with incident CVD outcomes of participants with CKM syndrome stages 0–3.

Outcome	Crude		Model 1		Model 2		Model 3	
	HR (95% CI)	*p*-value	HR (95% CI)	*p*-value	HR (95% CI)	*p*-value	HR (95% CI)	*p*-value
Cardiovascular disease								
Physical frailty								
Per 1 score increase	1.30 (1.28–1.31)	<0.001	1.32 (1.31–1.33)	<0.001	1.25 (1.24–1.27)	<0.001	1.21 (1.20–1.22)	<0.001
Non-frail	1.00 (Ref)		1.00 (Ref)		1.00 (Ref)		1.00 (Ref)	
Prefrail	1.33 (1.31–1.35)	<0.001	1.36 (1.34–1.38)	<0.001	1.28 (1.26–1.31)	<0.001	1.23 (1.21–1.25)	<0.001
Frail	2.44 (2.34–2.53)	<0.001	2.56 (2.46–2.66)	<0.001	2.15 (2.06–2.24)	<0.001	1.90 (1.83–1.98)	<0.001
*P* for trend		<0.001		<0.001		<0.001		<0.001
Frailty index								
Per 0.1 score increase	1.53 (1.51–1.54)	<0.001	1.57 (1.55–1.59)	<0.001	1.48 (1.47–1.50)	<0.001	1.40 (1.38–1.42)	<0.001
Non-frail	1.00 (Ref)		1.00 (Ref)		1.00 (Ref)		1.00 (Ref)	
Prefrail	1.52 (1.50–1.55)	<0.001	1.54 (1.51–1.56)	<0.001	1.45 (1.43–1.48)	<0.001	1.37 (1.35–1.40)	<0.001
Frail	2.48 (2.40–2.55)	<0.001	2.67 (2.60–2.76)	<0.001	2.34 (2.27–2.41)	<0.001	2.06 (2.00–2.13)	<0.001
*P* for trend		<0.001		<0.001		<0.001		<0.001
Coronary heart disease								
Physical frailty								
Per 1 score increase	1.34 (1.33–1.36)	<0.001	1.37 (1.35–1.39)	<0.001	1.29 (1.27–1.30)	<0.001	1.23 (1.21–1.24)	<0.001
Non-frail	1.00 (Ref)		1.00 (Ref)		1.00 (Ref)		1.00 (Ref)	
Prefrail	1.42 (1.38–1.45)	<0.001	1.46 (1.42–1.49)	<0.001	1.35 (1.32–1.39)	<0.001	1.28 (1.24–1.31)	<0.001
Frail	2.68 (2.54–2.82)	<0.001	2.82 (2.68–2.98)	<0.001	2.27 (2.15–2.40)	<0.001	1.94 (1.83–2.05)	<0.001
*P* for trend		<0.001		<0.001		<0.001		<0.001
Frailty index								
Per 0.1 score increase	1.64 (1.62–1.66)	<0.001	1.71 (1.69–1.74)	<0.001	1.61 (1.58–1.63)	<0.001	1.49 (1.47–1.51)	<0.001
Non-frail	1.00 (Ref)		1.00 (Ref)		1.00 (Ref)		1.00 (Ref)	
Prefrail	1.65 (1.61–1.69)	<0.001	1.69 (1.65–1.74)	<0.001	1.59 (1.55–1.63)	<0.001	1.47 (1.43–1.51)	<0.001
Frail	2.96 (2.84–3.08)	<0.001	3.29 (3.16–3.42)	<0.001	2.81 (2.70–2.93)	<0.001	2.38 (2.28–2.48)	<0.001
*P* for trend		<0.001		<0.001		<0.001		<0.001
Stroke								
Physical frailty								
Per 1 score increase	1.25 (1.22–1.28)	<0.001	1.26 (1.22–1.29)	<0.001	1.20 (1.17–1.23)	<0.001	1.16 (1.13–1.19)	<0.001
Non-frail	1.00 (Ref)		1.00 (Ref)		1.00 (Ref)		1.00 (Ref)	
Prefrail	1.29 (1.23–1.35)	<0.001	1.29 (1.24–1.36)	<0.001	1.23 (1.17–1.29)	<0.001	1.18 (1.13–1.24)	<0.001
Frail	2.17 (1.96–2.40)	<0.001	2.17 (1.95–2.40)	<0.001	1.85 (1.66–2.06)	<0.001	1.65 (1.48–1.84)	<0.001
*P* for trend		<0.001		<0.001		<0.001		<0.001
Frailty index								
Per 0.1 score increase	1.34 (1.30–1.38)	<0.001	1.33 (1.29–1.37)	<0.001	1.26 (1.22–1.30)	<0.001	1.18 (1.14–1.22)	<0.001
Non-frail	1.00 (Ref)		1.00 (Ref)		1.00 (Ref)		1.00 (Ref)	
Prefrail	1.32 (1.26–1.38)	<0.001	1.28 (1.22–1.35)	<0.001	1.22 (1.16–1.28)	<0.001	1.15 (1.09–1.20)	<0.001
Frail	1.85 (1.70–2.00)	<0.001	1.88 (1.73–2.04)	<0.001	1.65 (1.51–1.79)	<0.001	1.44 (1.32–1.57)	<0.001
*P* for trend		<0.001		<0.001		<0.001		<0.001
Heart failure								
Physical frailty								
Per 1 score increase	1.54 (1.51–1.57)	<0.001	1.56 (1.53–1.59)	<0.001	1.43 (1.40–1.47)	<0.001	1.35 (1.32–1.38)	<0.001
Non-frail	1.00 (Ref)		1.00 (Ref)		1.00 (Ref)		1.00 (Ref)	
Prefrail	1.67 (1.60–1.75)	<0.001	1.70 (1.62–1.77)	<0.001	1.53 (1.47–1.60)	<0.001	1.43 (1.36–1.49)	<0.001
Frail	4.13 (3.82–4.47)	<0.001	4.26 (3.94–4.62)	<0.001	3.15 (2.90–3.43)	<0.001	2.59 (2.38–2.82)	<0.001
*P* for trend		<0.001		<0.001		<0.001		<0.001
Frailty index								
Per 0.1 score increase	1.76 (1.71–1.80)	<0.001	1.80 (1.76–1.85)	<0.001	1.63 (1.59–1.68)	<0.001	1.48 (1.44–1.52)	<0.001
Non-frail	1.00 (Ref)		1.00 (Ref)		1.00 (Ref)		1.00 (Ref)	
Prefrail	1.80 (1.72–1.89)	<0.001	1.79 (1.71–1.87)	<0.001	1.63 (1.56–1.70)	<0.001	1.47 (1.41–1.54)	<0.001
Frail	3.45 (3.23–3.69)	<0.001	3.67 (3.44–3.93)	<0.001	2.92 (2.73–3.13)	<0.001	2.37 (2.20–2.54)	<0.001
*P* for trend		<0.001		<0.001		<0.001		<0.001
Atrial fibrillation								
Physical frailty								
Per 1 score increase	1.22 (1.20–1.24)	<0.001	1.25 (1.23–1.27)	<0.001	1.21 (1.19–1.23)	<0.001	1.17 (1.15–1.19)	<0.001
Non-frail	1.00 (Ref)		1.00 (Ref)		1.00 (Ref)		1.00 (Ref)	
Prefrail	1.24 (1.20–1.27)	<0.001	1.27 (1.23–1.31)	<0.001	1.23 (1.19–1.26)	<0.001	1.18 (1.15–1.22)	<0.001
Frail	2.06 (1.93–2.20)	<0.001	2.19 (2.05–2.34)	<0.001	1.98 (1.85–2.12)	<0.001	1.77 (1.65–1.89)	<0.001
*P* for trend		<0.001		<0.001		<0.001		<0.001
Frailty index								
Per 0.1 score increase	1.40 (1.38–1.43)	<0.001	1.43 (1.40–1.46)	<0.001	1.39 (1.36–1.41)	<0.001	1.31 (1.29–1.34)	<0.001
Non-frail	1.00 (Ref)		1.00 (Ref)		1.00 (Ref)		1.00 (Ref)	
Prefrail	1.42 (1.38–1.46)	<0.001	1.41 (1.36–1.45)	<0.001	1.36 (1.32–1.40)	<0.001	1.29 (1.25–1.33)	<0.001
Frail	1.97 (1.87–2.07)	<0.001	2.11 (2.00–2.22)	<0.001	1.95 (1.85–2.06)	<0.001	1.74 (1.65–1.84)	<0.001
*P* for trend		<0.001		<0.001		<0.001		<0.001
Peripheral artery disease								
Physical frailty								
Per 1 score increase	1.36 (1.33–1.38)	<0.001	1.37 (1.35–1.40)	<0.001	1.28 (1.25–1.31)	<0.001	1.24 (1.21–1.26)	<0.001
Non-frail	1.00 (Ref)		1.00 (Ref)		1.00 (Ref)		1.00 (Ref)	
Prefrail	1.39 (1.33–1.44)	<0.001	1.41 (1.36–1.47)	<0.001	1.30 (1.25–1.35)	<0.001	1.25 (1.20–1.30)	<0.001
Frail	2.80 (2.59–3.03)	<0.001	2.90 (2.68–3.14)	<0.001	2.27 (2.09–2.46)	<0.001	2.03 (1.87–2.21)	<0.001
*P* for trend		<0.001		<0.001		<0.001		<0.001
Frailty index								
Per 0.1 score increase	1.63 (1.59–1.66)	<0.001	1.65 (1.62–1.69)	<0.001	1.52 (1.48–1.56)	<0.001	1.44 (1.41–1.48)	<0.001
Non-frail	1.00 (Ref)		1.00 (Ref)		1.00 (Ref)		1.00 (Ref)	
Prefrail	1.66 (1.59–1.72)	<0.001	1.65 (1.59–1.72)	<0.001	1.51 (1.45–1.57)	<0.001	1.44 (1.38–1.50)	<0.001
Frail	2.78 (2.61–2.96)	<0.001	2.92 (2.74–3.10)	<0.001	2.38 (2.23–2.54)	<0.001	2.13 (1.99–2.28)	<0.001
*P* for trend		<0.001		<0.001		<0.001		<0.001

Model 1 was adjusted age, sex, ethnicity. Model 2 was adjusted age, sex, ethnicity, education level, BMI, Townsend deprivation index, smoking status, alcohol consumption, and physical activity. Model 3 was further adjusted for hypertension and diabetes. CI, confidence interval; HR, hazard ratio.

For overall CVD, each one-score increase in physical frailty was associated with a 21% higher risk (HR: 1.21, 95% CI: 1.20–1.22), whereas each 0.1-unit increase in frailty index was associated with a 40% higher risk (HR: 1.40, 95% CI: 1.38–1.42). Compared with non-frail participants, frail individuals exhibited substantially higher risks of CVD for both physical frailty (HR: 1.90, 95% CI: 1.83–1.98) and frailty index (HR: 2.06, 95% CI: 2.00–2.13). Among the cardiovascular outcomes examined, the strongest associations were observed for HF (Table 2).

Restricted cubic spline analyses demonstrated significant positive dose–response relationships between physical frailty, frailty index, and cardiovascular outcomes (all *p*-overall <0.001). Overall associations were predominantly linear, although evidence of nonlinearity was observed for selected outcomes.

### Incidence of cardiovascular outcomes

**Figure 4:**
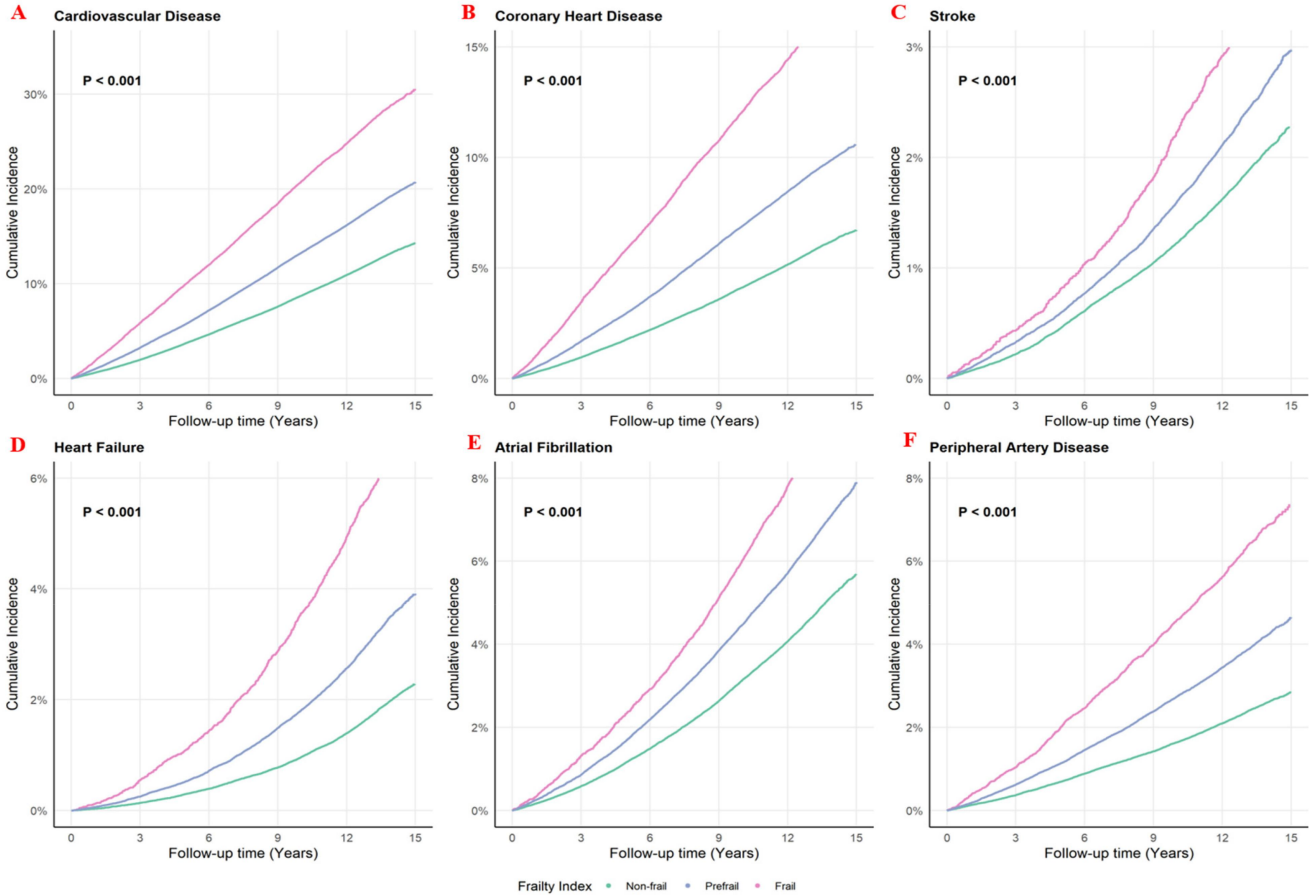
Cumulative incidence curves for cardiovascular disease outcomes stratified by frailty index. Participants with frailty exhibited progressively higher cumulative incidences across all cardiovascular outcomes. (Gray's test *p* < 0.001). **(A)** Cardiovascular disease; **(B)** coronary heart disease; **(C)** stroke; **(D)** heart failure; **(E)** atrial fibrillation; **(F)** peripheral artery disease.

The incidence of cardiovascular outcomes according to physical frailty and frailty index is summarized in Supplementary Tables S11,S12, and cumulative incidence curves are presented in Figure 4 and Supplementary Figure S2. Across both frailty assessment approaches, incidence rates of CVD and all major cardiovascular subtypes, including CHD, stroke, HF, AF, and PAD, progressively increased with increasing frailty severity (all *p* < 0.001). Frail participants consistently exhibited the highest incidence rates across all cardiovascular outcomes.

Similarly, cumulative incidence analyses demonstrated a stepwise increase in cardiovascular risk across frailty categories (Gray's test, all *p* < 0.001). Separation of cumulative incidence curves became more pronounced during follow-up, particularly for overall CVD and HF.

### Incremental predictive value of physical frailty and frailty index

The incremental predictive performance of physical frailty and frailty index beyond conventional risk factors is shown in Table 3. The addition of both physical frailty and frailty index significantly improved discrimination for CVD and all cardiovascular outcomes (all *p* < 0.001). Compared with physical frailty, frailty index generally demonstrated greater improvements in predictive performance across most outcomes. For overall CVD, incorporation of frailty index yielded a larger increase in Harrell's *C*-statistic than physical frailty (Δ*C*: 0.0093 vs. 0.0039). Similar patterns were observed for CHD, AF, and PAD, whereas the greatest improvement in predictive performance for both measures was observed for HF.

**Table 3:** Incremental discrimination of frailty status beyond conventional risk factors assessed by Harrell's C-statistic.

Outcome	C-statistic, model 2	C-statistic, model 3	Δ*C* (95%CI)	*p* value
Cardiovascular disease				
Physical frailty (per 1 unit)	0.7108	0.7147	0.0039 (0.0034–0.0043)	<0.001
Frailty index (per 0.1 unit)	0.7109	0.7202	0.0093 (0.0087–0.0099)	<0.001
Coronary heart disease				
Physical frailty (per 1 unit)	0.7245	0.7289	0.0044 (0.0038–0.0051)	<0.001
Frailty index (per 0.1 unit)	0.7245	0.737	0.0126 (0.0114–0.0138)	<0.001
Stroke				
Physical frailty (per 1 unit)	0.7163	0.7186	0.0023 (0.0016–0.0032)	<0.001
Frailty index (per 0.1 unit)	0.7163	0.7185	0.0022 (0.0014–0.0029)	<0.001
Heart failure				
Physical frailty (per 1 unit)	0.7736	0.7813	0.0077 (0.0065–0.0090)	<0.001
Frailty index (per 0.1 unit)	0.7738	0.7831	0.0094 (0.0081–0.0107)	<0.001
Atrial fibrillation				
Physical frailty (per 1 unit)	0.7445	0.7469	0.0023 (0.0019–0.0028)	<0.001
Frailty index (per 0.1 unit)	0.7444	0.7494	0.0050 (0.0042–0.0058)	<0.001
Peripheral artery disease				
Physical frailty (per 1 unit)	0.6988	0.7036	0.0048 (0.0038–0.0060)	<0.001
Frailty index (per 0.1 unit)	0.6993	0.7111	0.0118 (0.0103–0.0137)	<0.001

Model 2 was adjusted age, sex, ethnicity, education level, BMI, Townsend deprivation index, smoking status, alcohol consumption, and physical activity. Model 3 was further adjusted hypertension and diabetes. CI, confidence Interval.

### Subgroup analyses

**Figure 5:**
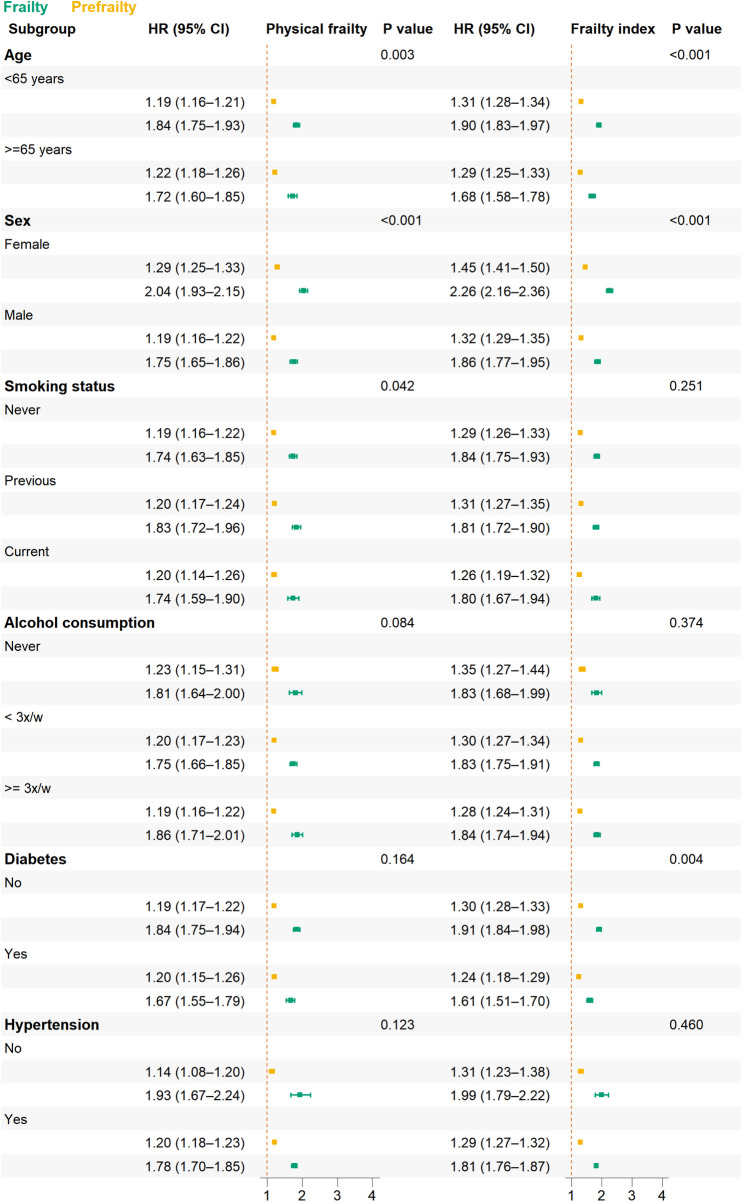
Subgroup analyses of frailty status and incident CVD. HRs and 95% CIs for incident CVD across frailty status are shown for subgroups defined by age, sex, smoking status, alcohol consumption, hypertension, and diabetes. CVD, cardiovascular disease; HR, hazard ratio; CI, confidence interval.

Subgroup analyses of the associations between physical frailty, frailty index, and incident cardiovascular outcomes are presented in Figure 5 and Supplementary Figures S3–S5. Overall, the positive associations between physical frailty, frailty index, and incident CVD outcomes remained generally consistent across subgroups defined by age, sex, smoking status, alcohol consumption, hypertension, and diabetes.

Significant effect modification was observed for several subgroup variables. Stronger associations were generally observed among younger individuals (<65 years) and women for both physical frailty and frailty index. Physical frailty showed significant interactions with age and sex for CVD, CHD, and AF, whereas frailty index demonstrated significant interactions for age, sex, and diabetes in selected outcomes. Nevertheless, the overall direction of associations remained consistent across all subgroup analyses.

### Sensitivity analyses

Results of the sensitivity analyses are presented in Supplementary Tables S13–S15. Time-dependent Cox regression analyses demonstrated that the associations of physical frailty and frailty index with incident CVD and its major subtypes remained significant across different follow-up intervals (0–5, 5–10, and >10 years), although the magnitude of associations showed slight attenuation during longer follow-up periods.

Similarly, the 2-year landmark analysis, which excluded participants who developed cardiovascular events within the first two years of follow-up, yielded results comparable to the primary analyses. Both physical frailty and frailty index remained significantly associated with increased risks of CVD and all major cardiovascular outcomes, with consistent dose–response relationships across frailty categories.

E-value analyses further suggested that the observed associations were relatively robust to potential unmeasured confounding. For overall CVD, E-values for point estimates ranged from 1.71 for physical frailty to 2.15 for frailty index, indicating that an unmeasured confounder would require a relatively strong association with both exposure and outcome to fully explain the observed findings.

To address potential overlap between frailty index construction and CKM staging criteria, we conducted sensitivity analyses using a modified 42-item frailty index after excluding seven cardiometabolic and cardiovascular-related deficits. The associations between the modified frailty index and incident CVD remained consistent with the primary analyses. Each 0.1-unit increase in modified frailty index was associated with a 35% higher risk of incident CVD after multivariable adjustment (HR, 1.35; 95% CI, 1.34–1.37). Similar associations were observed for CHD, stroke, HF, AF, and PAD (Supplementary Table S17).

The multiple-imputation analysis included an average of 444,774 participants, compared with 330,197 participants in the complete-case analysis. Baseline characteristics were generally comparable between the two analytic cohorts, and the incidence of CVD was similar (Supplementary Table S18). In the sensitivity analysis using multiple imputation, the associations of both physical frailty and the frailty index with incident CVD remained statistically significant and were directionally consistent with the complete-case findings. In Model 2, each one-point increase in the physical frailty score was associated with a higher risk of incident cardiovascular disease in both the complete-case analysis [HR: 1.25, 95% CI: 1.24–1.27] and the multiple-imputation analysis [HR: 1.21, 95% CI: 1.20–1.22]. Compared with non-frail participants, the HRs for prefrailty were 1.28 (95% CI: 1.26–1.31) and 1.24 (95% CI: 1.22–1.26), respectively, while the corresponding HRs for frailty were 2.15 (95% CI: 2.06–2.24) and 1.89 (95% CI: 1.82–1.96). All *P* values for trend were <0.001. Similar findings were observed for the frailty index. Each 0.1-unit increase in the frailty index was associated with incident cardiovascular disease in the complete-case analysis [HR: 1.48, 95% CI: 1.47–1.50] and the multiple-imputation analysis [HR: 1.44, 95% CI: 1.42–1.45]. Compared with non-frail participants, the HRs for prefrailty were 1.45 (95% CI: 1.43–1.48) and 1.41 (95% CI: 1.38–1.43), respectively, and the HRs for frailty were 2.34 (95% CI: 2.27–2.41) and 2.13 (95% CI: 2.08–2.19), respectively. All *P* values for trend were <0.001. Although the effect estimates were modestly attenuated after multiple imputation, the direction, dose-response pattern, and statistical significance of the associations remained unchanged, supporting the robustness of the primary findings (Supplementary Table S19).

In the competing-risk analysis, the associations of physical frailty and the frailty index with incident CVD remained significant after accounting for death as a competing event. In Model 2, each one-point increase in the physical frailty score was associated with a 19.4% higher subdistribution hazard of incident CVD (sHR, 1.194; 95% CI, 1.181–1.206). Compared with non-frail participants, prefrail and frail participants had sHRs of 1.210 (95% CI, 1.188–1.232) and 1.818 (95% CI, 1.744–1.896), respectively. Each 0.1-unit increase in the frailty index was associated with an sHR of 1.416 (95% CI, 1.399–1.433). Compared with the non-frail group, the corresponding sHRs were 1.391 (95% CI, 1.365–1.417) for prefrailty and 2.094 (95% CI, 2.028–2.162) for frailty. significant graded associations were observed for both frailty measures, with all *p* values for trend <0.001 (Supplementary Table S20).

### CKM stage-stratified analyses

To examine whether the observed associations were primarily driven by participants with CKM stage 3, additional analyses were performed separately across CKM stages 0–3. The associations of both physical frailty and frailty index with incident CVD were observed across all CKM stages (Supplementary Table S16). For physical frailty, each one-score increase was associated with higher CVD risk in CKM stage 0, stage 1, stage 2, and stage 3, with adjusted HRs of 1.30 (95% CI, 1.18–1.43), 1.15 (95% CI, 1.08–1.23), 1.27 (95% CI, 1.25–1.29), and 1.21 (95% CI, 1.19–1.23), respectively. Compared with non-frail participants, frail participants had higher incident CVD risk across CKM stage 0, stage 1, stage 2, and stage 3, with adjusted HRs of 3.15 (95% CI, 2.04–4.86), 2.32 (95% CI, 1.76–3.07), 2.18 (95% CI, 2.06–2.31), and 1.93 (95% CI, 1.82–2.05), respectively. Prefrailty was also associated with higher CVD risk in CKM stage 0, stage 2, and stage 3, whereas the association was attenuated in stage 1 after full adjustment.

Similar associations were observed for frailty index. Each 0.1-unit increase in frailty index was associated with increased CVD risk across CKM stage 0, stage 1, stage 2, and stage 3, with adjusted HRs of 1.45 (95% CI, 1.30–1.62), 1.45 (95% CI, 1.35–1.56), 1.49 (95% CI, 1.46–1.51), and 1.41 (95% CI, 1.39–1.43), respectively. Compared with non-frail participants, frail participants defined by frailty index also showed consistently higher CVD risk across CKM stage 0, stage 1, stage 2, and stage 3, with adjusted HRs of 2.25 (95% CI, 1.58–3.21), 1.99 (95% CI, 1.59–2.51), 2.32 (95% CI, 2.22–2.42), and 2.14 (95% CI, 2.04–2.24), respectively. Significant dose-dependent trends were observed across frailty categories in all CKM stages. These findings suggest that the observed associations were not restricted to participants with CKM stage 3.

## Discussion

In this large prospective cohort study of individuals with CKM stages 0–3, we observed that both physical frailty and frailty index were independently associated with increased risks of incident CVD and its major subtypes, including CHD, stroke, HF, AF, and PAD. Clear dose-response relationships were identified, with progressively increasing cardiovascular risks across worsening frailty categories. Importantly, although both measures showed robust predictive ability, frailty index generally provided superior incremental predictive performance compared with physical frailty. These findings remained consistent across subgroup and sensitivity analyses.

Our findings extend previous evidence linking frailty with adverse cardiovascular and metabolic outcomes. To our knowledge, this is the first study within the UK Biobank cohort to simultaneously evaluate the associations of both physical frailty and frailty index with incident cardiovascular disease and its major subtypes among individuals with CKM stages 0–3. By focusing on individuals with CKM stages 0–3, our study provides evidence regarding cardiovascular risk before clinically overt cardiovascular disease and highlights the potential role of frailty assessment in cardiovascular risk stratification across the CKM continuum.

Previous studies have primarily linked frailty to CKM progression, cardiovascular mortality, and metabolic complications. For example, among individuals with type 2 diabetes, both pre-frailty and frailty have been associated with increased risks of diabetic microvascular complications and cardiovascular mortality (8, 9, 11, 18–20). In addition, recent studies suggest that biological aging indicators, including frailty status, are associated with accelerated CKM progression (19). However, evidence regarding incident cardiovascular outcomes across the CKM continuum, particularly using different frailty constructs within the same population, remains limited. Our findings extend these observations by demonstrating that frailty predicts future cardiovascular events in individuals with CKM stages 0–3, representing a clinically relevant period within the CKM continuum before clinically overt cardiovascular disease.

The observed associations may be explained by several shared biological mechanisms. CKM syndrome and frailty are characterized by substantial pathophysiological overlap, with chronic low-grade inflammation potentially representing a central pathway linking frailty to adverse cardiovascular outcomes (8, 21). Persistent inflammatory activation has been increasingly recognized as an important contributor to frailty development and disease progression (22). Chronic inflammation may accelerate physiological deterioration across multiple organ systems, reduce physiological reserve, and increase vulnerability to cardiovascular injury. Supporting this hypothesis, a recent UK Biobank study reported that inflammatory markers were associated with elevated frailty levels and that frailty partially mediated inflammation-related disease risk (23). Collectively, these findings suggest that persistent inflammatory activity may contribute to the development of frailty and subsequently increase susceptibility to adverse cardiovascular outcomes.

An important finding of our study was that frailty index generally provided greater incremental predictive value than physical frailty. This difference may be attributable to their distinct conceptual frameworks. Physical frailty primarily reflects biological and functional decline through physical manifestations such as weakness, exhaustion, slow gait speed, and reduced activity (24). In contrast, frailty index captures the multidimensional accumulation of health deficits across multiple physiological systems, including comorbidities, functional impairment, and psychosocial characteristics (6). Recent proteomic evidence further supports the notion that frailty reflects broader aging-related biological processes involving inflammatory pathways and cellular senescence (19). Therefore, frailty index may provide a more comprehensive representation of cumulative biological aging and systemic deterioration.

The two frailty measures used in this study represent related but distinct frailty constructs. Physical frailty was defined according to the Fried phenotype framework and therefore primarily reflects physical manifestations of reduced physiological reserve. In contrast, the frailty index was constructed according to the Rockwood cumulative deficit principle. Because the Rockwood model is a conceptual framework rather than a fixed list of variables, the specific deficits included in frailty indices may vary across cohorts depending on data availability (15). Our UK Biobank-based frailty index differed from some other Rockwood-style indices by using available UK Biobank health deficits, including sensory, psychological, cardiometabolic, respiratory, musculoskeletal, pain, cancer, and gastrointestinal domains. Nevertheless, it remains conceptually comparable with deficit-accumulation frailty indices because it quantifies frailty as the proportion of accumulated health deficits.

These findings should be interpreted as supporting the value of frailty as a complementary risk marker rather than as evidence that frailty is a causal determinant of CVD. Our findings also have important clinical implications. CKM stages 0–3 provide a critical opportunity for preventive intervention before overt cardiovascular disease develops. Traditional cardiovascular risk assessment largely focuses on cardiometabolic risk factors, whereas frailty assessment may identify vulnerable individuals who are not fully captured by conventional models. Importantly, frailty should not be viewed as an irreversible condition. Sustained frailty remission has been associated with lower subsequent CVD risk, suggesting that frailty itself may be a modifiable target. Additionally, healthy lifestyle behaviors, dietary protein intake, and improved vitamin D status have been associated with favorable frailty transitions and reduced adverse outcomes (25, 26). These observations suggest that integrating frailty assessment into CKM management may facilitate more targeted preventive strategies.

In subgroup analyses, the associations of both physical frailty and frailty index with incident CVD appeared stronger among participants aged <65 years and among women. This finding is partly consistent with the study by Verschoor et al., who analyzed 161,149 community-dwelling adults from Ontario, Canada, and reported that frailty was associated with increased mortality and healthcare utilization across adulthood, with the frailty-related hazard of death being greater at younger ages (27). With respect to sex differences, our finding of stronger frailty-CVD associations among women is consistent with Verschoor et al., who reported that frailty-related mortality risk was particularly pronounced among younger women (27). However, it differs from the study by Lu et al., which found that phenotypic frailty was an independent predictor of mortality in men but not women in the I-Lan Longitudinal Aging Study (28). Lu et al. also reported sex-specific differences in body composition among frail individuals: frail women had higher BMI and waist circumference than non-frail women, whereas frail men had lower BMI and reduced appendicular skeletal muscle mass. These findings suggest that frailty may represent different biological and cardiometabolic vulnerability profiles in men and women. In our CKM population, where metabolic abnormalities and adiposity-related risk are central components of disease progression, frailty among women may capture a particularly adverse cardiometabolic phenotype. Sapp et al., in a systematic review and meta-analysis of laboratory-based frailty indices, found no consistent evidence of sex differences in frailty index -Lab scores, although frailty index -Lab was associated with mortality and other adverse health outcomes. These differences indicate that sex-specific frailty associations may vary according to the frailty instrument, study population, age distribution, and outcome examined (29). Therefore, although our findings suggest stronger frailty-CVD associations among women and younger participants, these subgroup results should be interpreted cautiously and require further validation. The stronger associations observed among women may partly reflect sex-related differences in the body-composition phenotype accompanying frailty. Lu et al. (28). reported that frail women had higher BMI and waist circumference than non-frail women, whereas frail men were characterized by lower BMI and appendicular skeletal muscle mass. Similarly, Soh and Won (30) found that fat-related indices, including body fat percentage and trunk fat mass index, were associated with poor physical performance in women, while low fat-free mass was the predominant correlate in men. Li et al. (31). further reported that frailty severity was positively correlated with estimated visceral fat area in women but not in men. These findings suggest that adiposity-related pathways may be particularly relevant to frailty-related vulnerability in some women. However, the evidence is not consistent, as Bunch et al. found that CT-derived visceral adiposity and lower skeletal muscle density were associated with frailty in men but not in women (32). Therefore, sex-specific body composition may provide a possible biological context for our findings, but whether differences in fat distribution explain the stronger frailty–CVD association among women remains uncertain.

An interesting finding from the CKM stage-stratified analyses was that the relative associations between frailty and incident CVD appeared stronger among individuals with CKM stage 0 than among those with CKM stage 3. This pattern should be interpreted cautiously and in the context of differences in baseline absolute risk. Individuals with CKM stage 3 already have subclinical cardiovascular disease or very-high-risk kidney disease, and their cardiovascular risk is likely driven by multiple established pathological pathways. Therefore, the additional relative prognostic contribution of frailty may be attenuated in later CKM stages, reflecting a potential ceiling effect. In contrast, among individuals with CKM stage 0, frailty may capture reduced physiological reserve, accumulated biological vulnerability, or unrecognized multisystem dysfunction that is not reflected by conventional cardiometabolic risk factors. Thus, frailty may have greater relative prognostic value when CKM-related risk is otherwise low, whereas in advanced CKM stages it may represent one contributor among multiple established risk factors. Several additional factors may also explain the stage-specific pattern. First, the number of CVD events, particularly among frail participants, was smaller in CKM stages 0 and 1 than in stages 2 and 3, which may have contributed to wider confidence intervals and less stable relative risk estimates. Second, survivor bias may have attenuated relative associations in CKM stage 3, because individuals with both advanced CKM abnormalities and severe frailty may have developed clinical CVD or died before baseline and therefore were not included in the analytic cohort. Third, residual heterogeneity within CKM stages may persist, as individuals classified within the same CKM stage may still differ in underlying metabolic, renal, inflammatory, subclinical cardiovascular, and functional profiles. Taken together, these findings suggest that frailty assessment should be interpreted as a complementary tool for cardiovascular risk stratification within the CKM continuum rather than as a standalone screen for CKM severity.

Several strengths and limitations should be considered. Major strengths include the large sample size, prospective design, long-term follow-up, simultaneous evaluation of both physical frailty and frailty index, and comprehensive analyses across multiple cardiovascular outcomes. In addition, extensive subgroup analyses and sensitivity analyses confirmed the robustness of our findings. However, several limitations warrant consideration. First, the UK Biobank primarily includes individuals of European ancestry, which may limit generalizability to other populations. Second, frailty status was assessed only at baseline, and longitudinal changes in frailty status were unavailable. Third, despite extensive adjustment and E-value analyses, residual confounding cannot be completely excluded. Finally, although participants with clinically manifest CVD were excluded at baseline, reverse causality and disease overlap cannot be completely excluded. Particularly among individuals with CKM stage 3, frailty may partly reflect underlying subclinical cardiovascular or kidney abnormalities rather than representing an entirely independent risk marker. Although 2-year landmark analyses and CKM stage-stratified analyses showed consistent associations, these approaches cannot fully eliminate the possibility of bidirectional interactions between ageing, frailty, CKM progression, and cardiovascular disease. Although some overlap between frailty-related health deficits and CKM components is inevitable in cumulative deficit models, sensitivity analyses excluding major cardiometabolic and cardiovascular-related deficits yielded similar results, supporting the robustness of the association between frailty and cardiovascular risk.

## Conclusion

In conclusion, among individuals with CKM stages 0–3, both physical frailty and frailty index were independently associated with increased risks of incident CVD and its major cardiovascular subtypes. Frailty index generally provided greater incremental predictive value beyond conventional risk factors. As the first UK Biobank study evaluating these complementary frailty constructs across CKM stages 0–3, our findings suggest that frailty assessment may provide complementary information for cardiovascular risk stratification within the CKM continuum, particularly by identifying vulnerable individuals not fully captured by conventional CKM-related risk factors. Frailty should therefore be interpreted as a complementary risk marker rather than a direct indicator of CKM severity.

## Data Availability

This research was conducted using the UK Biobank Resource under Application Number 1099804. Data are available to researchers upon application to the UK Biobank (https://www.ukbiobank.ac.uk/) and cannot be redistributed by the authors due to licensing restrictions.
