# Supplementary material for: Frailty for cardiovascular risk stratification in CKM syndrome stages 0–3: a UK Biobank prospective cohort study
Source: Front Cardiovasc Med. 2026 Aug 5;13:1918446. doi: 10.3389/fcvm.2026.1918446 (PMC13485804; doi:10.3389/fcvm.2026.1918446)
Supplement: Supplementary file 1 [file Datasheet1.docx]

### Supplementary material

**Supplementary Table S1. Pattern of missingness across model covariates.**

**Supplementary Table S2.** Unit conversion factors used in the calculation.

**Supplementary Table S3.** Definitions of cardiovascular disease in the UK Biobank.

**Supplementary Table S4.** Definitions of CKM conditions.

**Supplementary Table S5.** The PREVENT 10-year risk estimation model equations.

**Supplementary Table S6.** Detailed definition of CKM syndrome.

**Supplementary Table S7.** Definition of physical frailty in the UK Biobank

**Supplementary Table S8.** The frailty items and scoring for construction of the frailty index.

**Supplementary Table S9.** Baseline characteristics of the participants in accordance with physical frailty.

**Supplementary Table S10.** Baseline characteristics of study participants according to CKM stage.

**Supplementary Table S11.** Incidence of cardiovascular outcomes according to physical frailty.

**Supplementary Table S12.** Incidence of cardiovascular outcomes according to frailty index.

**Supplementary Table S13.** Time-dependent Cox regression analyses of the association between frailty status and incident CVD outcomes across follow-up intervals.

**Supplementary Table S14.** Two-year landmark analysis of the association between frailty status and incident CVD outcomes.

**Supplementary Table S15.** E-value analysis for quantifying the potential impact of unmeasured confounding on primary associations.

**Supplementary Table S16.** Associations of physical frailty and frailty index with incident cardiovascular disease stratified by CKM stage.

**Supplementary Table S17.** Sensitivity analyses using a modified frailty index excluding cardiometabolic and cardiovascular-related deficits for the association with incident cardiovascular outcomes.

**Supplementary Table S18.** Comparison of baseline characteristics between the complete-case and multiple-imputation analyses

**Supplementary Table 19**. Comparison of the associations of physical frailty and frailty index with incident CVD between complete-case and multiple-imputation analyses.

**Supplementary Table S20.** Associations of physical frailty and frailty index with incident CVD using Fine–Gray competing-risk models.

**Supplementary Figure S1.** Dose–response associations of physical frailty with CVD outcomes.

**Supplementary Figure S2**. Cumulative incidence curves for CVD outcomes stratified by physical frailty.

**Supplementary Figure S3.** Subgroup analyses of frailty status and incident CHD.

**Supplementary Figure S4.** Subgroup analyses of frailty status and incident heart failure.

**Supplementary Figure S5.** Subgroup analyses of frailty status and incident atrial fibrillation.

**Supplementary Table S1.** Pattern of missingness across model covariates.

| **Covariate** | **Missing (n)** | **Missing (%)** |
| --- | --- | --- |
| Age | 18 | 0.004 |
| Sex | 18 | 0.004 |
| Ethnicity | 918 | 0.183 |
| WC | 2181 | 0.434 |
| Standing height | 2559 | 0.509 |
| BMI | 3125 | 0.622 |
| DBP | 34458 | 6.859 |
| SBP | 34445 | 6.856 |
| TC | 33297 | 6.627 |
| Glucose | 72932 | 14.516 |
| Serum Creatinine | 33154 | 6.599 |
| TG | 33297 | 6.628 |
| HbA1c | 36009 | 7.168 |
| HDL-C | 72628 | 14.457 |

Abbreviations: TC, Total Cholesterol; HDL-C, High-Density Lipoprotein Cholesterol; SBP, Systolic Blood Pressure; DBP, Diastolic Blood Pressure; BMI, Body Mass Index; WC, Waist Circumference; HbA1c, Hb (Hemoglobin) A1c; Scr, Serum Creatinine; TG, Triglycerides

**Supplementary Table S2.** Unit conversion factors used in the calculation.

| **Measurement** | **SI Unit** | **Unit Used in Formula** | **Conversion Factor** | **Notes** |
| --- | --- | --- | --- | --- |
| Glucose | mmol/L | mg/dL | × 18.02 | Used in CKM calculation |
| Creatinine (Cr) | µmol/L | mg/dL | ÷ 88.4 | Used in CKM calculation |
| Hb (Hemoglobin) A1c (HbA1c) | mmol/mol | % | × 0.09148 + 2.152 | Used in CKM calculation |
| High-Density Lipoprotein (HDL) | mmol/L | mg/dL | × 38.67 | Used in CKM calculatio |
| Low-Density Lipoprotein (LDL) | mmol/L | mg/dL | × 38.67 | Used in CKM calculatio |
| Total Cholesterol (TC) | mmol/L | mg/dL | × 38.67 | Used in CKM calculatio |

**Supplementary Table S3.** Definitions of cardiovascular disease in the UK Biobank.

| **Cardiovascular disease** | **Data field ID** | **ICD-10** |
| --- | --- | --- |
| Coronary heart disease | 131296; 131298; 131300; 131304; 131306 | I20.0;I20.1;I20.8;I20.9; I21.0; I21.1; I21.2; I21.3; I21.4; I21.9; I22.0; I22.1; I22.8; I22.9; I24.0; I24.1; I24.8; I24.9; I25.0; I25.1; I25.2; I25.3; I25.4; I25.5; I25.6; I25.8; I25.9; |
| Stroke | 131360; 131362; 131364; 131366; 131368 | I60.0; I60.1; I60.2; I60.3; I60.4; I60.5; I60.6; I60.7; I60.8; I60.9; I61.0; I61.1; I61.2; I61.3; I61.4; I61.5; I61.6; I61.8; I61.9; I62.1; I63.0; I63.1; I63.2; I63.3; I63.4; I63.5; I63.6; I63.8; I63.9; I64; |
| Heart failure | 131354 | I50.0; I50.1; I50.9; |
| Atrial fibrillation | 131350 | I48.0; I48.1; I48.2; I48.3; I48.4; I48.9 |
| Peripheral Artery Disease | 131380; 131382; 131384; 131386; 131388; 131390; 131392; 131394 | I70.0; I70.00; I70.01; I70.1; I70.10; I70.11; I70.2; I70.20; I70.21; I70.8; I70.80; I70.81; I70.9; I70.90; I70.91; I71.0; I71.1; I71.2; I71.3; I71.4; I71.5; I71.6; I71.8; I71.9; I72.0; I72.1; I72.2; I72.3; I72.4; I72.5; I72.6; I72.8; I72.9; I73.0; I73.1; I73.8; I73.9; I74.0; I74.1; I74.2; I74.3; I74.4; I74.5; I74.8; I74.9; I77.1; I77.2; I77.3; I77.4; I77.5; I77.6; I77.8; I77.9; I78.0; I78.1; I78.8; I78.9; I79.1; I79.2; I79.8 |

Abbreviation: International Statistical Classification of Diseases and Related Health Problems, 10th Revision

**Supplementary Table S4.** Definitions of CKM conditions.

| **CKM conditions** | **Definition** | **CKM indicators** | **Threshold for CKM indicators** |
| --- | --- | --- | --- |
| CVD | Individuals with clinical CVD or subclinical CVD | Clinical CVD | History of chronic heart failure, coronary heart disease, peripheral artery disease, atrial fibrillation, or stroke |
|  |  | Subclinical CVD | Any of the following criterion is met:   - Participants with advanced-stage CKD include those with an eGFR < 30 mL/min/1.73 m^2^. - Predicted 10-year CVD risk ≥ 20% (PREVENT equations) |
| Kidney  diseases | Individuals with CKD | Moderate-to-high-risk CKD | The moderate to high-risk CKD was defined as an estimated Glomerular Filtration Rate (eGFR) between 30 and 60 mL/min/1.73 m2, and eGFR was calculated using the CKD-EPI 2021 creatinine-based equation proposed by the Chronic Kidney Epidemiology Collaboration. |
|  |  | Very high-risk CKD | Very high-risk CKD in KDIGO classification: Participants with advanced-stage CKD include those with an eGFR < 30 mL/min/1.73 m^2^. |
| Metabolic disorders | Individuals with overweight/obesity, abdominal obesity, prediabetes,  diabetes, hypertension,  hypertriglyceridemia or MetS | Overweight obesity | BMI ≥ 25 kg/m^2^ (or ≥ 23 kg/m^2^ if Asian ancestry) |
|  |  | Abdominal obesity | Waist circumference ≥ 88/102 cm in female/male (or if Asian ancestry ≥ 80/90 cm in female/male) |
|  |  | Prediabetes | Fasting blood glucose ≥ 100-124 mg/dL or HbA1c ≥ 5.7%-6.4% and without self-reported diagnosis of diabetes, use of insulin, or oral hypoglycemic agents |
|  |  | Diabetes | Fasting blood glucose ≥ 125 mg/dL or HbA1c ≥ 6.5% or self-reported diagnosis of diabetes, use of insulin, or oral hypoglycemic agents |
|  |  | Hypertension | SBP ≥ 130 mm Hg or DBP ≥ 80 mmHg or self-reported diagnosis of hypertension or use of antihypertensive medications |
|  |  | Hypertriglyceridemia | TG ≥ 135 mg/dl or self-reported physician-diagnosed, and/or current use of lipid-lowering drugs |
|  |  | MetS | MetS is defined by the presence of 3 or more of the following:   - Abdominal obesity. - HDL cholesterol <40 mg/dL for men and <50 mg/dL for women - TG ≥150 mg/dL - elevated blood pressure (SBP ≥130 mmHg or DBP ≥80 mmHg or use of antihypertensive medications - Fasting blood glucose ≥ 100 mg/dL |

Abbreviations: BMI, body mass index; CKD, chronic kidney disease; CKM, cardiovascular-kidney-metabolic; CVD, cardiovascular disease; DBP, diastolic blood pressure; eGFR, estimated glomerular filtration rate; HDL, high-density lipoprotein; KDIGO, The Kidney Disease: Improving Global Outcomes; MetS, metabolic syndrome; SBP, systolic blood pressure; UACR, urinary albumin to creatinine ratio.

10-year cardiovascular risk was estimated with the AHA Predicting Risk of CVD EVENTs (PREVENT) equations. High risk was defined as ≥20%, 10-year CVD risk (based on recommended thresholds [https://professional.heart.org/en/guidelines and-statements/prevent-calculator]).

**Supplementary Table S5.** The PREVENT 10-year risk estimation model equations.

| **Sex** | **Calculation** |
| --- | --- |
| Men | log-Odds = –3.031168 + 0.7688528 × (age – 55) /10 + 0.0736174 × ((TC – HDL-C) × 0.02586 – 3.5) – 0.0954431 × (HDL-C × 0.02586 – 1.3) /0.3 – 0.4347345 × (min(SBP, 110) – 110) /20 + 0.3362658 × (max(SBP, 110) – 130) /20 + 0.7692857 × (if diabetes) + 0.4386871 × (if current smoker) + 0.5378979 × (min(eGFR, 60) – 60) / -15 + 0.0164827 × (max(eGFR, 60) – 90) / -15 + 0.288879 × (if using anti-hypertensive medication) – 0.1337349 × (if using statin) – 0.0475924 × (if using anti-hypertensive medication) × (max(SBP, 110) – 130) /20 + 0.150273 × (if using statin) × ((TC – HDL-C) × 0.02586 – 3.5) – 0.0517874 × (age – 55) /10 × ((TC – HDL-C) × 0.02586 – 3.5) + 0.0191169 × (age – 55) /10 × (HDL-C × 0.02586 – 1.3) /0.3 – 0.1049477 × (age – 55) /10 × (max(SBP, 110) – 130) /20 – 0.2251948 × (age – 55) /10 × (if diabetes) – 0.0895067 × (age – 55) /10 × (if current smoker) – 0.1543702 × (age – 55) /10 × (min(eGFR, 60) – 60) / –15 |
|  | **Risk** = 1 / (1 + exp(-log-Odds)) |
| Women | log-Odds = -3.307728 + 0.7939329 × (age – 55) /10 + 0.0305239 × ((TC – HDL-C) × 0.02586 – 3.5) – 0.1606857 × (HDL-C × 0.02586 – 1.3) /0.3 – 0.2394003 × (min(SBP, 110) – 110) /20 + 0.360078 × (max(SBP, 110) – 130) /20 + 0.8667604 × (if diabetes) + 0.5360739 × (if current smoker) + 0.6045917 × (min(eGFR, 60) – 60) / -15 + 0.0433769 × (max(eGFR, 60) – 90) / -15 + 0.3151672 × (if using anti-hypertensive medication) – 0.1477655 × (if using statin) – 0.0663612 × (if using anti-hypertensive medication) × (max(SBP, 110) – 130) /20 + 0.1197879 × (if using statin) × ((TC – HDL-C) × 0.02586 – 3.5) – 0.0819715 × (age – 55) /10 × ((TC – HDL-C) × 0.02586 – 3.5) + 0.0306769 × (age – 55) /10 × (HDL-C × 0.02586 – 1.3) /0.3 – 0.0946348 × (age – 55) /10 × (max(SBP, 110) – 130) /20 – 0.27057 × (age – 55) /10 × (if diabetes) – 0.078715 × (age – 55) /10 × (if current smoker) – 0.1637806 × (age – 55) /10 × (min(eGFR, 60) – 60) / –15 |
|  | **Risk** = 1 / (1 + exp(–log-Odds)) |

Abbreviations: TC, Total Cholesterol; HDL-C, High-Density Lipoprotein Cholesterol; SBP, Systolic Blood Pressure; eGFR, estimated Glomerular Filtration Rate; PREVENT, Predicting Risk of Cardiovascular Disease EVENTs; CVD, Cardiovascular Disease; CKD, Chronic Kidney Disease; CKM, Cardiovascular-Kidney-Metabolic; KDIGO, Kidney Disease: Improving Global Outcomes; UACR, Urine Albumin-to-Creatinine Ratio

**Supplementary Table S6.** Detailed definition of CKM syndrome.

| **Stages** | **Definition** |
| --- | --- |
| **Stage 0:** No CKM risk factors | Stage 0 is defined as the absence of risk factors for cardiovascular-kidney-metabolic (CKM) syndrome, including normal body mass index (BMI), waist circumference (WC), blood glucose, systolic blood pressure (SBP), diastolic blood pressure (DBP), lipid levels, renal function, and no evidence of cardiovascular disease (CVD). |
| **Stage 1:** Excess or dysfunctional adiposity | Excess or dysfunctional adiposity (any one of the following):   1. Identified individuals with an elevated BMI (> 23 kg/m^2^ for individuals with Asian race and > 25 kg/m^2^ for all other race and ethnic groups) 2. An elevated waist circumference (≥ 80 and≥ 90 cm for women and men with Asian race, respectively, and ≥ 88 and ≥ 102 cm for women and men in other race and ethnicity categories, respectively) 3. Prediabetes (defined as a glycated hemoglobin of 5.7% to < 6.5% or a fasting blood glucose of 100 mg/dL to < 126 mg/dL). |
| **Stage 2:** Metabolic risk factors and CKD | Any of the five criteria is met:   1. Hypertriglyceridemia is defined by a self-reported physician-diagnosed, and/or current use of lipid-lowering drugs, and/or TG ≥ 135 mg/dl 2. Hypertension is defined by SBP ≥ 130 mmHg, DBP ≥ 80 mmHg, a medical diagnosis, or taking antihypertensive medication. 3. Diabetes is defined by FBG > 126 mg/dL, HbAc ≥ 6.5%, and/or a self-reported physician-diagnosed, and/or taking hypoglycemic agents 4. Metabolic syndrome is defined as meeting at least three of the following criteria: (1) An elevated waist circumference (≥ 80 and≥ 90 cm for women and men with Asian race, respectively, and ≥ 88 and ≥ 102 cm for women and men in other race and ethnicity categories, respectively); (2) reduced high-density lipoprotein cholesterol (HDL-C < 40 mg/dL in men or < 50 mg/dL in women); (3) high TG (TG > 150 mg/dL); (4) SBP/DBP ≥ 130/80 mmHg and/or use of antihypertensive medications; or (5) Fasting blood glucose ≥ 100 mg/dL 5. Moderate-to-high-risk CKD |
| **Stage 3:** Subclinical CVD in CKM | Individuals with subclinical CVD or advanced-stage CKD.  1. Very high-risk CKD in KDIGO classification: eGFR < 30 ml/min/1.73m^2^.   2. High predicted 10-year CVD risk: the predicted risk ≥20%, as calculated by the PREVENT equations. |

Abbreviations: TC, Total Cholesterol; HDL-C, High-Density Lipoprotein Cholesterol; SBP, Systolic Blood Pressure; DBP, Diastolic Blood Pressure; eGFR, estimated Glomerular Filtration Rate; PREVENT, Predicting Risk of Cardiovascular Disease EVENTs; CVD, Cardiovascular Disease; CKD, Chronic Kidney Disease; CKM, Cardiovascular-Kidney-Metabolic; KDIGO, Kidney Disease: Improving Global Outcomes; UACR, Urine Albumin-to-Creatinine Ratio; BMI, Body Mass Index; WC, Waist Circumference; KDIGO, Kidney Disease: Improving Global Outcomes; HbA1c, Hb (Hemoglobin) A1c.

**Supplementary Table S7.** Definition of physical frailty in the UK Biobank

| **Components** | **Definition**^*^ | **Field IDs in UK Biobank** |
| --- | --- | --- |
| **Weight loss** | **Question**: “Compared with one year ago, has your weight changed?”  **Answer**: Yes, lost weight = 1; other = 0. | 2306 |
| **Exhaustion** | **Question**: “Over the past two weeks, how often have you felt tired or had little energy?”  **Answer**: More than half the days or nearly every day = 1; other = 0. | 2080 |
| **Low physical activity** | **Question**: “In the last 4 weeks did you spend any time doing the following? (You can select more than one answer)”  **Answer**: Walking for pleasure (not as a means of transport) = 0; other exercises (eg: swimming, cycling, keep fit, bowling) = 0; strenuous sports = 0; light DIY (eg: pruning, watering the lawn): frequency of once per week or less=1; frequency of more than once per week=0; heavy DIY (eg: weeding, lawn mowing, carpentry, digging) = 0; none of the above = 1; | 6164, 1011 |
| **Slow gait speed** | **Question**: “How would you describe your usual walking pace?”  **Answer**: Slow = 1; other = 0. | 924 |
| **Low grip strength** | **Definition**: Grip strength (kg) was lower than sex- and BMI-specific cut-off points.  **Cut-off points**: **Men**: BMI ≤24.0 kg/m^2^, grip strength ≤29 kg; 24.1< BMI ≤28.0 kg/m^2^, grip strength ≤30 kg; BMI >28.0 kg/m^2^, grip strength ≤32 kg;  **Women**: BMI ≤23.0 kg/m^2^, grip strength ≤17 kg; 23.1< BMI ≤26.0 kg/m^2^, grip strength ≤17.3 kg; 26.1< BMI ≤29.0 kg/m^2^, grip strength ≤18 kg; BMI >29.0 kg/m^2^, grip strength ≤21 kg | 31, 21001, 46, 47 |
| **Pre-frailty or frailty** | A composite score was calculated by summing above five components, ranging from 0 to 5.  Participants were classified as non-frail (the composite score: 0), pre-frail (the composite score: 1-2), or frail (the composite score: ≥ 3). |  |

* Answer of “prefer not to answer” to each component was treated as missing data. *Abbreviation*: BMI body mass index.

**Supplementary Table S8.** The frailty items and scoring for construction of the frailty index.

| **Type of deficit** | **Item** | **Trait** | **UK Biobank data field** | **Coded in frailty index** |
| --- | --- | --- | --- | --- |
| Sensory | 1 | Glaucoma * | 6148; 20002(1277) | Yes = 1; No = 0; Prefer not to answer = NA |
|  | 2 | Cataracts * | 6148; 20002(1278) | Yes = 1; No = 0; Prefer not to answer = NA |
|  | 3 | Hearing difficulty | 2247 | Yes = 1; I am completely deaf = 1; No = 0;  Do not know = NA; Prefer not to answer = NA |
| Cranial | 4 | Migraine | 20002(1265) | Yes-you will be asked about this later by an interviewer = 1;  No = 0; Do not know = NA; Prefer not to answer = NA |
|  | 5 | Dental problems | 6149 | Mouth ulcers = 1; Painful gums = 1; Bleeding gums = 1; Loose teeth = 1; Toothache = 1; Dentures = 1;  None of the above = 0; Prefer not to answer = NA |
| Mental wellbeing | 6 | Self-rated health | 2178 | Poor = 1; Fair = 0.5; Good = 0.25; Excellent = 0;  Do not know = NA; Prefer not to answer = NA |
|  | 7 | Fatigue: frequency of tiredness / lethargy in last two weeks | 2080 | Nearly every day = 1; More than half the days = 0.5; Several days = 0.25; Not at all = 0; Do not know = NA; Prefer not to answer = NA |
|  | 8 | Sleep: experience of sleeplessness/insomnia | 1200 | Usually = 1; Sometimes = 0.5; Never/rarely = 0; Prefer not to answer = NA |
|  | 9 | Depressed feelings: frequency in last two weeks | 2050 | Nearly every day = 1; More than half the days = 0.75; Several days = 0.5; Not at al1 = 0; Do not know = NA; Prefer not to answer = NA |
|  | 10 | Self-described nervous personality | 1970 | Yes = 1; No = 0; Do not know = NA; Prefer not to answer = NA |
|  | 11 | Severe anxiety/ panic attacks | 20002(1287) | Yes-you will be asked about this later by an interviewer = 1;  No = 0; Do not know = NA; Prefer not to answer = NA |
|  | 12 | Common to feel loneliness | 2020 | Yes = 1; No = 0; Do not know = NA; Prefer not to answer = NA |
|  | 13 | Sense of misery (ever/never) | 1930 | Yes = 1; No = 0; Do not know = NA; Prefer not to answer = NA |
| Infirmity | 14 | Infirmity: long-standing illness or disability | 2188 | Yes = 1; No = 0; Do not know = NA; Prefer not to answer = NA |
|  | 15 | Falls in last year | 2296 | More than one fall = 1; Only one fal1 = 0.5;  No falls = 0; Prefer not to answer = NA |
|  | 16 | Fractures/broken bones in last five years | 2463 | Yes = 1; No = 0; Do not know = NA; Prefer not to answer = NA |
| Cardiometabolic | 17 | Diabetes | 2443; 20002(1220&1223) | Yes = 1; No = 0; Do not know = NA; Prefer not to answer = NA |
|  | 18 | Heart attack* | 6150; 20002(1075) | Yes = 1; No = 0; Prefer not to answer = NA |
|  | 19 | Angina * | 6150; 20002(1074) | Yes = 1; No = 0; Prefer not to answer = NA |
|  | 20 | Stroke * | 6150; 20002(1081&1583) | Yes = 1; No = 0; Prefer not to answer = NA |
|  | 21 | High blood pressure* | 6150; 20002(1065) | Yes = 1; No = 0; Prefer not to answer = NA |
|  | 22 | Hypothyroidism * | 20002(1226) | Yes-you will be asked about this later by an interviewer = 1;  No = 0; Do not know = NA; Prefer not to answer = NA |
|  | 23 | Blood clot in the leg (DVT) * | 6152; 20002(1904) | Yes = 1; No = 0; Prefer not to answer = NA |
|  | 24 | Cholesterol lowering medication * | 6153 | Yes = 1; No = 0; Prefer not to answer = NA |
| Respiratory | 25 | Breathing: wheeze in last year | 2316 | Yes = 1; No = 0; Do not know = NA; Prefer not to answer = NA |
|  | 26 | Pneumonia | 20002(1398) | Yes-you will be asked about this later by an interviewer = 1;  No = 0; Do not know = NA; Prefer not to answer = NA |
|  | 27 | Emphysema/chronic bronchitis * | 6152; 20002(1113) | Yes = 1; No = 0; Prefer not to answer = NA |
|  | 28 | Asthma * | 6152; 20002(1111) | Yes = 1; No = 0; Prefer not to answer = NA |
| Musculoskeletal | 29 | Rheumatoid arthritis | 20002(1464) | Yes-you will be asked about this later by an interviewer = 1;  No = 0; Do not know = NA; Prefer not to answer = NA |
|  | 30 | Osteoarthritis | 20002(1465) | Yes-you will be asked about this later by an interviewer = 1;  No = 0; Do not know = NA; Prefer not to answer = NA |
|  | 31 | Gout | 20002(1466) | Yes-you will be asked about this later by an interviewer = 1  No = 0; Do not know = NA; Prefer not to answer = NA |
|  | 32 | Osteoporosis | 20002(1309) | Yes-you will be asked about this later by an interviewer = 1;  No = 0; Do not know = NA; Prefer not to answer = NA |
| Immunological | 33 | Hayfever, allergic rhinitis or eczema * | 6152; 20002(1387&1452) | Yes = 1; No = 0; Prefer not to answer = NA |
|  | 34 | Psoriasis | 20002(1453) | Yes-you will be asked about this later by an interviewer = 1;  No = 0; Do not know = NA; Prefer not to answer = NA |
| Cancer | 35 | Any cancer diagnosis | 2453; 134 | Yes-you will be asked about this later by an interviewer = 1;  No = 0; Do not know = NA; Prefer not to answer = NA |
|  | 36 | Multiple cancers diagnosed | 134 | More than 1 = 1; 0 or 1 = 0 |
| Pain | 37 | Chest pain | 2335 | Yes = 1; No = 0; Do not know = NA; Prefer not to answer = NA |
|  | 38 | Headache * | 6159 | Yes = 1; No = 0; Prefer not to answer = NA |
|  | 39 | Facial pain * | 6159 | Yes = 1; No = 0; Prefer not to answer = NA |
|  | 40 | Neck or shoulder pain * | 6159 | Yes = 1; No = 0; Prefer not to answer = NA |
|  | 41 | Back pain * | 6159 | Yes = 1; No = 0; Prefer not to answer = NA |
|  | 42 | Stomach or abdominal pain * | 6159 | Yes = 1; No = 0; Prefer not to answer = NA |
|  | 43 | Hip pain * | 6159 | Yes = 1; No = 0; Prefer not to answer = NA |
|  | 44 | Knee pain * | 6159 | Yes = 1; No = 0; Prefer not to answer = NA |
|  | 45 | Sciatica | 20002(1476) | Yes-you will be asked about this later by an interviewer = 1;  No = 0; Do not know = NA; Prefer not to answer = NA |
| Gastrointestina | 46 | Gastric reflux | 20002(1138) | Yes-you will be asked about this later by an interviewer = 1;  No = 0; Do not know = NA; Prefer not to answer = NA |
|  | 47 | Hiatus hernia | 20002(1474) | Yes-you will be asked about this later by an interviewer = 1;  No = 0; Do not know = NA; Prefer not to answer = NA |
|  | 48 | Gall stones | 20002(1462) | Yes-you will be asked about this later by an interviewer = 1;  No = 0; Do not know = NA; Prefer not to answer = NA |
|  | 49 | Diverticulitis | 20002(1458) | Yes-you will be asked about this later by an interviewer = 1;  No = 0; Do not know = NA; Prefer not to answer = NA |

Variables included in frailty index were adapted from the articles[1,2].

* Participants reported medically diagnosed conditions for these items.

1.	Zhong Q, Zhou R, Huang Y-N, Huang R-D, Li F-R, Chen H-W, Wei Y-F, Liu K, Cao B-F, Liao K-Y et al: Frailty and risk of metabolic dysfunction-associated steatotic liver disease and other chronic liver diseases. J Hepatol 2024, 82(3):427–437.

2.	Clegg A, Bates C, Young J, Ryan R, Nichols L, Ann Teale E, Mohammed MA, Parry J, Marshall T: Development and validation of an electronic frailty index using routine primary care electronic health record data. Age Ageing 2016, 45(3):353–360.

**Supplementary Table S9**. Baseline characteristics of the participants in accordance with physical frailty.

| **Characteristic** | **Physical frailty** | | | |  |
| --- | --- | --- | --- | --- | --- |
|  | **Overall** | **Non-frail** | **Prefrail** | **Frail** | ***p*** |
| **N** | 330197 | 194567 | 124084 | 9819 |  |
| **Demographic** |  |  |  |  |  |
| Age, years, mean (SD) | 56.08 (8.09) | 55.93 (8.06) | 56.23 (8.14) | 57.09 (7.76) | <0.001 |
| Gender, female, (%) | 148030 (44.8) | 91710 (47.1) | 52177 (42.0) | 3357 (34.2) | <0.001 |
| Ethnicity, White, n (%) | 311416 (94.3) | 187063 (96.1) | 114677 (92.4) | 8463 (86.2) | <0.001 |
| Education level, n (%) |  |  |  |  | <0.001 |
| Low | 90250 (27.3) | 45584 (23.4) | 38884 (31.3) | 4715 (48.0) |  |
| Intermediate | 130274 (39.5) | 77279 (39.7) | 49168 (39.6) | 3425 (34.9) |  |
| High | 109673 (33.2) | 71704 (36.9) | 36032 (29.0) | 1679 (17.1) |  |
| Townsend deprivation index (mean (SD)) | -1.39 (3.02) | -1.71 (2.84) | -1.04 (3.16) | 0.36 (3.51) | <0.001 |
| **Lifestyle factor** |  |  |  |  |  |
| Smoking status, n (%) |  |  |  |  | <0.001 |
| Never | 183842 (55.9) | 111251 (57.3) | 66881 (54.1) | 4800 (49.3) |  |
| Former | 111087 (33.8) | 65690 (33.9) | 41868 (33.9) | 3107 (31.9) |  |
| Current | 34034 (10.3) | 17100 (8.8) | 14834 (12.0) | 1834 (18.8) |  |
| Alcohol consumption, n (%) |  |  |  |  | <0.001 |
| Never | 24712 (7.5) | 10450 (5.4) | 11783 (9.5) | 2040 (20.8) |  |
| < 3 times/week | 159462 (48.3) | 87988 (45.2) | 64995 (52.4) | 5647 (57.5) |  |
| ≥ 3 times/week | 146023 (44.2) | 96129 (49.4) | 47306 (38.1) | 2132 (21.7) |  |
| Sleep duration, n (%) |  |  |  |  | <0.001 |
| < 6 h | 18848 (5.7) | 7565 (3.9) | 9104 (7.3) | 1782 (18.1) |  |
| 6–9 h | 306099 (92.7) | 185200 (95.2) | 112339 (90.5) | 7333 (74.7) |  |
| >9 h | 5250 (1.6) | 1802 (0.9) | 2641 (2.1) | 704 (7.2) |  |
| Physical activity, n (%) |  |  |  |  | <0.001 |
| Low | 111406 (33.7) | 55470 (28.5) | 48018 (38.7) | 6501 (66.2) |  |
| Moderate | 109386 (33.1) | 71481 (36.7) | 36627 (29.5) | 1139 (11.6) |  |
| High | 109405 (33.1) | 67616 (34.8) | 39439 (31.8) | 2179 (22.2) |  |
| **Clinical characteristic** |  |  |  |  |  |
| Body Mass Index, kg/m², (mean (SD)) | 27.23 (4.66) | 26.47 (4.10) | 28.11 (4.99) | 30.78 (6.56) | <0.001 |
| Systolic blood pressure, mmHg, (mean (SD)) | 137.74 (18.56) | 137.90 (18.61) | 137.47 (18.45) | 137.55 (18.59) | <0.001 |
| Diastolic blood pressure, mmHg, (mean (SD)) | 82.42 (10.06) | 82.41 (10.06) | 82.38 (10.05) | 82.79 (10.17) | 0.001 |
| Diabetes, n (%) | 34830 (10.5) | 14352 (7.4) | 17346 (14.0) | 2732 (27.8) | <0.001 |
| Hypertension, n (%) | 251413 (76.1) | 145651 (74.9) | 96050 (77.4) | 8254 (84.1) | <0.001 |
| Dyslipidemia, n (%) | 184140 (55.8) | 102220 (52.5) | 73825 (59.5) | 6958 (70.9) | <0.001 |
| Incident cardiovascular disease, n (%) | 52454 (15.9) | 27057 (13.9) | 22106 (17.8) | 2874 (29.3) | <0.001 |
| Incident coronary heart disease, n (%) | 26289 (8.0) | 13031 (6.7) | 11448 (9.2) | 1583 (16.1) | <0.001 |
| Incident stroke, n (%) | 7604 (2.3) | 3961 (2.0) | 3192 (2.6) | 400 (4.1) | <0.001 |
| Incident atrial fibrillation, n (%) | 19610 (5.9) | 10443 (5.4) | 8045 (6.5) | 990 (10.1) | <0.001 |
| Incident heart failure, n (%) | 8790 (2.7) | 3909 (2.0) | 4059 (3.3) | 735 (7.5) | <0.001 |
| Incident peripheral artery disease, n (%) | 10975 (3.3) | 5442 (2.8) | 4711 (3.8) | 705 (7.2) | <0.001 |
| **Laboratory parameter** |  |  |  |  |  |
| Triglycerides, mg/dl, median (IQR) | 130.38 [91.85, 188.83] | 126.39 [89.28, 183.16] | 134.98 [95.12, 194.94] | 151.90 [106.37, 215.93] | <0.001 |
| Glucose, mg/dl, median (IQR) | 88.65 [82.80, 95.38] | 88.52 [82.75, 94.93] | 88.78 [82.82, 95.83] | 90.05 [83.41, 99.69] | <0.001 |
| Creatinine, mg/dl, median (IQR) | 0.79 [0.69, 0.91] | 0.80 [0.70, 0.92] | 0.78 [0.68, 0.90] | 0.76 [0.66, 0.88] | <0.001 |
| HDL-C, mg/dl, (mean (SD)) | 56.61 (14.75) | 57.79 (14.89) | 55.17 (14.38) | 52.14 (13.98) | <0.001 |
| Total Cholesterol mg/dl, (mean (SD)) | 223.12 (42.93) | 225.06 (42.03) | 220.75 (43.71) | 215.70 (47.46) | <0.001 |

Abbreviation: HDL-C, high-density lipoprotein cholesterol; SD, Standard Deviation; IQR, Interquartile Range.

**Supplementary Table S10.** Baseline characteristics of study participants according to CKM stage.

| **Characteristic** | **Overall** | **Stage 0** | **Stage 1** | **Stage 2** | **Stage 3** | ***p* value** |
| --- | --- | --- | --- | --- | --- | --- |
| N | 330197 | 15814 | 31005 | 161414 | 121964 |  |
| **Demographic** |  |  |  |  |  |  |
| Age, years, mean (SD) | 56.08 (8.09) | 50.78 (7.42) | 52.01 (7.66) | 58.00 (7.49) | 55.25 (8.21) | <0.001 |
| Gender, female, (%) | 148030 (44.8) | 2918 (18.5) | 7526 (24.3) | 34777 (21.5) | 102809 (84.3) | <0.001 |
| Ethnicity, White, n (%) | 311416 (94.3) | 14566 (92.1) | 29694 (95.8) | 152791 (94.7) | 114365 (93.8) | <0.001 |
| Education level, n (%) |  |  |  |  |  | <0.001 |
| Low | 90250 (27.3) | 2349 (14.9) | 5984 (19.3) | 48165 (29.8) | 33752 (27.7) |  |
| Intermediate | 130274 (39.5) | 5676 (35.9) | 12373 (39.9) | 63655 (39.4) | 48570 (39.8) |  |
| High | 109673 (33.2) | 7789 (49.3) | 12648 (40.8) | 49594 (30.7) | 39642 (32.5) |  |
| Townsend deprivation index (mean (SD)) | -1.39 (3.02) | -1.35 (3.04) | -1.44 (2.96) | -1.48 (2.96) | -1.26 (3.11) | <0.001 |
| **Lifestyle factor** |  |  |  |  |  |  |
| Smoking status, n (%) |  |  |  |  |  | <0.001 |
| Never | 183842 (55.9) | 10135 (64.2) | 18513 (59.9) | 94314 (58.7) | 60880 (50.1) |  |
| Former | 111087 (33.8) | 4138 (26.2) | 9533 (30.8) | 53366 (33.2) | 44050 (36.3) |  |
| Current | 34034 (10.3) | 1503 (9.5) | 2885 (9.3) | 13088 (8.1) | 16558 (13.6) |  |
| Alcohol consumption, n (%) |  |  |  |  |  | <0.001 |
| Never | 24712 (7.5) | 1187 (7.5) | 1887 (6.1) | 13093 (8.1) | 8545 (7.0) |  |
| < 3 times/week | 159462 (48.3) | 7530 (47.6) | 16238 (52.4) | 78865 (48.9) | 56829 (46.6) |  |
| ≥ 3 times/week | 146023 (44.2) | 7097 (44.9) | 12880 (41.5) | 69456 (43.0) | 56590 (46.4) |  |
| Sleep duration, n (%) |  |  |  |  |  | <0.001 |
| < 6 h | 306099 (92.7) | 15098 (95.5) | 29277 (94.4) | 148971 (92.3) | 112753 (92.4) |  |
| 6–9 h | 18848 (5.7) | 576 (3.6) | 1392 (4.5) | 9883 (6.1) | 6997 (5.7) |  |
| >9 h | 5250 (1.6) | 140 (0.9) | 336 (1.1) | 2560 (1.6) | 2214 (1.8) |  |
| Physical activity, n (%) |  |  |  |  |  | <0.001 |
| Low | 109405 (33.1) | 5524 (34.9) | 10634 (34.3) | 52824 (32.7) | 40423 (33.1) |  |
| Moderate | 109386 (33.1) | 5927 (37.5) | 11224 (36.2) | 52453 (32.5) | 39782 (32.6) |  |
| High | 111406 (33.7) | 4363 (27.6) | 9147 (29.5) | 56137 (34.8) | 41759 (34.2) |  |
| **Clinical characteristic** |  |  |  |  |  |  |
| Body Mass Index, kg/m², (mean (SD)) | 27.23 (4.66) | 21.22 (1.41) | 25.74 (3.03) | 27.01 (4.72) | 28.68 (4.40) | <0.001 |
| Waist circumference, cm, (mean (SD)) | 89.73 (13.21) | 72.12 (5.97) | 83.14 (9.34) | 86.79 (12.10) | 97.58 (11.29) | <0.001 |
| Systolic blood pressure, mmHg, (mean (SD)) | 137.74 (18.56) | 114.85 (8.47) | 117.15 (7.75) | 141.49 (17.41) | 140.98 (17.37) | <0.001 |
| Diastolic blood pressure, mmHg, (mean (SD)) | 82.42 (10.06) | 70.32 (5.67) | 71.97 (5.15) | 83.57 (9.19) | 85.11 (9.78) | <0.001 |
| Diabetes, n (%) | 34830 (10.5) | 0 (0.0) | 0 (0.0) | 16725 (10.4) | 18105 (14.8) | <0.001 |
| Hypertension, n (%) | 251413 (76.1) | 0 (0.0) | 0 (0.0) | 147430 (91.3) | 103983 (85.3) | <0.001 |
| Dyslipidemia, n (%) | 184140 (55.8) | 0 (0.0) | 0 (0.0) | 67174 (41.6) | 116966 (95.9) | <0.001 |
| Incident cardiovascular disease, n (%) | 52417 (15.9) | 747 (4.7) | 1700 (5.5) | 24264 (15.0) | 25706 (21.1) | <0.001 |
| Incident coronary heart disease, n (%) | 26266 (8.0) | 183 (1.2) | 480 (1.5) | 10533 (6.5) | 15070 (12.4) | <0.001 |
| Incident stroke, n (%) | 7586 (2.3) | 99 (0.6) | 208 (0.7) | 3804 (2.4) | 3475 (2.8) | <0.001 |
| Incident atrial fibrillation, n (%) | 19605 (5.9) | 258 (1.6) | 665 (2.1) | 9652 (6.0) | 9030 (7.4) | <0.001 |
| Incident heart failure, n (%) | 8783 (2.7) | 64 (0.4) | 170 (0.5) | 4165 (2.6) | 4384 (3.6) | <0.001 |
| Incident peripheral artery disease, n (%) | 10971 (3.3) | 262 (1.7) | 484 (1.6) | 4996 (3.1) | 5229 (4.3) | <0.001 |
| **Laboratory parameter** |  |  |  |  |  |  |
| Triglycerides, mg/dl, median (IQR) | 130.38 [91.85, 188.83] | 78.74 [63.42, 98.05] | 89.01 [70.77, 108.50] | 113.19 [85.74, 151.19] | 188.74 [148.62, 253.84] | <0.001 |
| Glucose, mg/dl, median (IQR) | 88.65 [82.80, 95.38] | 84.44 [79.13, 89.35] | 86.49 [80.95, 92.12] | 89.28 [83.61, 95.83] | 89.06 [82.82, 96.57] | <0.001 |
| Creatinine, mg/dl, median (IQR) | 0.79 [0.69, 0.91] | 0.72 [0.65, 0.81] | 0.75 [0.67, 0.85] | 0.75 [0.66, 0.85] | 0.88 [0.78, 0.98] | <0.001 |
| Cystatin C, median (IQR) | 0.88 [0.80, 0.97] | 0.79 [0.73, 0.86] | 0.82 [0.76, 0.90] | 0.87 [0.79, 0.96] | 0.92 [0.84, 1.00] | <0.001 |
| Glycated hemoglobin (%), (median [IQR]) | 5.36 [5.13, 5.59] | 5.21 [5.02, 5.39] | 5.25 [5.04, 5.45] | 5.37 [5.15, 5.60] | 5.39 [5.17, 5.65] | <0.001 |
| Total Cholesterol, mg/dl, (mean (SD)) | 223.12 (42.93) | 207.20 (36.18) | 212.31 (36.84) | 224.42 (41.98) | 226.20 (45.48) | <0.001 |
| **Frailty status** |  |  |  |  |  |  |
| Physical frailty score (mean (SD)) | 0.56 (0.80) | 0.38 (0.65) | 0.47 (0.70) | 0.58 (0.82) | 0.58 (0.81) | <0.001 |
| Physical frailty category (%) |  |  |  |  |  | <0.001 |
| Non-frail | 194567 (59.2) | 11006 (69.8) | 19538 (63.2) | 93314 (58.1) | 70709 (58.3) |  |
| Prefrail | 124084 (37.8) | 4580 (29.0) | 10909 (35.3) | 61916 (38.6) | 46679 (38.5) |  |
| Frail | 9819 (3.0) | 181 (1.1) | 475 (1.5) | 5310 (3.3) | 3853 (3.2) |  |
| Frailty index score (mean (SD)) | 0.12 (0.07) | 0.10 (0.06) | 0.10 (0.06) | 0.12 (0.07) | 0.12 (0.07) | <0.001 |
| Frailty index category (%) |  |  |  |  |  | <0.001 |
| Non-frail | 194969 (59.0) | 11197 (70.8) | 21005 (67.7) | 91263 (56.5) | 71504 (58.6) |  |
| Prefrail | 116688 (35.3) | 4263 (27.0) | 9092 (29.3) | 59925 (37.1) | 43408 (35.6) |  |
| Frail | 18540 (5.6) | 354 (2.2) | 908 (2.9) | 10226 (6.3) | 7052 (5.8) |  |

Abbreviation: SD, Standard Deviation; IQR, Interquartile Range

**Supplementary Table S11.** Incidence of cardiovascular outcomes according to physical frailty.

| **Outcome** | **Physical frailty** | | |  |
| --- | --- | --- | --- | --- |
|  | **Non-frail** | **Prefrail** | **Frail** | ***p* value** |
| **Cardiovascular Disease** |  |  |  |  |
| Events, n (%) | 27040 (13.9) | 22088 (17.8) | 2872 (29.2) | <0.001 |
| Incidence rate, /1,000 person-years | 10.97 | 14.5 | 26.23 |  |
| **Coronary Heart Disease** |  |  |  |  |
| Events, n (%) | 13023 (6.7) | 11437 (9.2) | 1579 (16.1) | <0.001 |
| Incidence rate, /1,000 person-years | 5.13 | 7.24 | 13.56 |  |
| **Stroke** |  |  |  |  |
| Events, n (%) | 3953 (2.0) | 3182 (2.6) | 400 (4.1) | <0.001 |
| Incidence rate, /1,000 person-years | 1.52 | 1.95 | 3.24 |  |
| **Heart Failure** |  |  |  |  |
| Events, n (%) | 3906 (2.0) | 4055 (3.3) | 735 (7.5) | <0.001 |
| Incidence rate, /1,000 person-years | 1.5 | 2.49 | 6.01 |  |
| **Atrial Fibrillation** |  |  |  |  |
| Events, n (%) | 10439 (5.4) | 8044 (6.5) | 990 (10.1) | <0.001 |
| Incidence rate, /1,000 person-years | 4.08 | 5.01 | 8.22 |  |
| **Peripheral Artery Disease** |  |  |  |  |
| Events, n (%) | 5440 (2.8) | 4709 (3.8) | 705 (7.2) | <0.001 |
| Incidence rate, /1,000 person-years | 2.11 | 2.91 | 5.81 |  |

**Supplementary Table S12.** Incidence of cardiovascular outcomes according to frailty index.

| **Outcome** | **Frailty index** | | |  |
| --- | --- | --- | --- | --- |
|  | **Non-frail** | **Prefrail** | **Frail** | ***p* value** |
| **Cardiovascular Disease** |  |  |  |  |
| Events, n (%) | 25171 (12.9) | 21993 (18.8) | 5253 (28.3) | <0.001 |
| Incidence rate, /1,000 person-years | 10.16 | 15.4 | 24.8 |  |
| **Coronary Heart Disease** |  |  |  |  |
| Events, n (%) | 11856 (6.1) | 11355 (9.7) | 3055 (16.5) | <0.001 |
| Incidence rate, /1,000 person-years | 4.65 | 7.65 | 13.63 |  |
| **Stroke** |  |  |  |  |
| Events, n (%) | 3901 (2.0) | 3028 (2.6) | 657 (3.5) | <0.001 |
| Incidence rate, /1,000 person-years | 1.5 | 1.97 | 2.75 |  |
| **Heart Failure** |  |  |  |  |
| Events, n (%) | 3708 (1.9) | 3924 (3.4) | 1151 (6.2) | <0.001 |
| Incidence rate, /1,000 person-years | 1.43 | 2.56 | 4.84 |  |
| **Atrial Fibrillation** |  |  |  |  |
| Events, n (%) | 9764 (5.0) | 8112 (7.0) | 1729 (9.3) | <0.001 |
| Incidence rate, /1,000 person-years | 3.81 | 5.38 | 7.4 |  |
| **Peripheral Artery Disease** |  |  |  |  |
| Events, n (%) | 4933 (2.5) | 4800 (4.1) | 1238 (6.7) | <0.001 |
| Incidence rate, /1,000 person-years | 1.91 | 3.15 | 5.26 |  |

**Supplementary Table S13.** Time-dependent Cox regression analyses of the association between frailty status and incident CVD outcomes across follow-up intervals.

| **Outcome** | **Follow-up interval** | **HR (95% CI)** | ***p* value** |  | **Follow-up interval** | **HR (95% CI)** | ***p* value** |
| --- | --- | --- | --- | --- | --- | --- | --- |
| **Cardiovascular Disease** | | | | | | | |
| Physical Frailty | 0-5 years | 1.22 (1.20-1.24) | <0.001 | Frailty Index | 0-5 years | 1.44 (1.41-1.47) | <0.001 |
|  | 5-10 years | 1.22 (1.20-1.24) | <0.001 |  | 5-10 years | 1.41 (1.38-1.44) | <0.001 |
|  | >10 years | 1.18 (1.16-1.21) | <0.001 |  | >10 years | 1.35 (1.32-1.38) | <0.001 |
| **Coronary Heart Disease** | | | | | | | |
| Physical Frailty | 0-5 years | 1.25 (1.22-1.28) | <0.001 | Frailty Index | 0-5 years | 1.55 (1.51-1.59) | <0.001 |
|  | 5-10 years | 1.24 (1.21-1.26) | <0.001 |  | 5-10 years | 1.49 (1.46-1.53) | <0.001 |
|  | >10 years | 1.19 (1.16-1.22) | <0.001 |  | >10 years | 1.43 (1.39-1.47) | <0.001 |
| **Stroke** | | | | | | | |
| Physical Frailty | 0-5 years | 1.16 (1.11-1.23) | <0.001 | Frailty Index | 0-5 years | 1.15 (1.08-1.23) | <0.001 |
|  | 5-10 years | 1.13 (1.09-1.18) | <0.001 |  | 5-10 years | 1.19 (1.13-1.25) | <0.001 |
|  | >10 years | 1.18 (1.13-1.23) | <0.001 |  | >10 years | 1.19 (1.13-1.25) | <0.001 |
| **Heart Failure** | | | | | | | |
| Physical Frailty | 0-5 years | 1.37 (1.30-1.44) | <0.001 | Frailty Index | 0-5 years | 1.48 (1.38-1.58) | <0.001 |
|  | 5-10 years | 1.39 (1.35-1.44) | <0.001 |  | 5-10 years | 1.55 (1.49-1.62) | <0.001 |
|  | >10 years | 1.31 (1.27-1.36) | <0.001 |  | >10 years | 1.43 (1.37-1.48) | <0.001 |
| **Atrial Fibrillation** | | | | | | | |
| Physical Frailty | 0-5 years | 1.13 (1.10-1.17) | <0.001 | Frailty Index | 0-5 years | 1.32 (1.27-1.37) | <0.001 |
|  | 5-10 years | 1.20 (1.17-1.23) | <0.001 |  | 5-10 years | 1.32 (1.28-1.36) | <0.001 |
|  | >10 years | 1.18 (1.15-1.21) | <0.001 |  | >10 years | 1.30 (1.26-1.34) | <0.001 |
| **Peripheral Artery Disease** | | | | | | | |
| Physical Frailty | 0-5 years | 1.25 (1.20-1.29) | <0.001 | Frailty Index | 0-5 years | 1.43 (1.37-1.50) | <0.001 |
|  | 5-10 years | 1.25 (1.21-1.30) | <0.001 |  | 5-10 years | 1.47 (1.41-1.53) | <0.001 |
|  | >10 years | 1.21 (1.17-1.26) | <0.001 |  | >10 years | 1.42 (1.36-1.48) | <0.001 |

Abbreviations: HR, hazard ratio; CI, confidence interval.

**Supplementary Table S14.** Two-year landmark analysis of the association between frailty status and incident CVD outcomes.

| **Outcome** | **Crude** | |  | **Model 1** | |  | **Model 2** | |  | **Model 3** | |
| --- | --- | --- | --- | --- | --- | --- | --- | --- | --- | --- | --- |
|  | **HR (95% CI)** | ***p*-value** |  | **HR (95% CI)** | ***p*-value** |  | **HR (95% CI)** | ***p*-value** |  | **HR (95% CI)** | ***p*-value** |
| **Cardiovascular Disease** |  |  |  |  |  |  |  |  |  |  |  |
| Physical Frailty |  |  |  |  |  |  |  |  |  |  |  |
| Continuous (per 1 score) | 1.29 (1.28-1.30) | <0.001 |  | 1.31 (1.30-1.33) | <0.001 |  | 1.25 (1.24-1.26) | <0.001 |  | 1.20 (1.19-1.22) | <0.001 |
| Non-frail | 1.00 (Ref) |  |  | 1.00 (Ref) |  |  | 1.00 (Ref) |  |  | 1.00 (Ref) |  |
| Prefrail | 1.32 (1.30-1.35) | <0.001 |  | 1.36 (1.33-1.38) | <0.001 |  | 1.28 (1.25-1.30) | <0.001 |  | 1.23 (1.20-1.25) | <0.001 |
| Frail | 2.38 (2.28-2.48) | <0.001 |  | 2.51 (2.40-2.61) | <0.001 |  | 2.10 (2.01-2.19) | <0.001 |  | 1.86 (1.78-1.94) | <0.001 |
| P for trend |  | <0.001 |  |  | <0.001 |  |  | <0.001 |  |  | <0.001 |
| Frailty Index |  |  |  |  |  |  |  |  |  |  |  |
| Per 0.1 score increase | 1.51 (1.49-1.53) | <0.001 |  | 1.55 (1.53-1.57) | <0.001 |  | 1.47 (1.45-1.48) | <0.001 |  | 1.38 (1.37-1.40) | <0.001 |
| Non-frail | 1.00 (Ref) |  |  | 1.00 (Ref) |  |  | 1.00 (Ref) |  |  | 1.00 (Ref) |  |
| Prefrail | 1.51 (1.48-1.54) | <0.001 |  | 1.52 (1.49-1.55) | <0.001 |  | 1.44 (1.41-1.47) | <0.001 |  | 1.36 (1.33-1.38) | <0.001 |
| Frail | 2.41 (2.34-2.49) | <0.001 |  | 2.61 (2.52-2.69) | <0.001 |  | 2.28 (2.21-2.36) | <0.001 |  | 2.00 (1.94-2.07) | <0.001 |
| P for trend |  | <0.001 |  |  | <0.001 |  |  | <0.001 |  |  | <0.001 |
| **Coronary Heart Disease** |  |  |  |  |  |  |  |  |  |  |  |
| Physical Frailty |  |  |  |  |  |  |  |  |  |  |  |
| Continuous (per 1 score) | 1.34 (1.32-1.36) | <0.001 |  | 1.37 (1.35-1.38) | <0.001 |  | 1.28 (1.27-1.30) | <0.001 |  | 1.22 (1.21-1.24) | <0.001 |
| Non-frail | 1.00 (Ref) |  |  | 1.00 (Ref) |  |  | 1.00 (Ref) |  |  | 1.00 (Ref) |  |
| Prefrail | 1.41 (1.38-1.45) | <0.001 |  | 1.45 (1.41-1.49) | <0.001 |  | 1.35 (1.31-1.39) | <0.001 |  | 1.27 (1.24-1.31) | <0.001 |
| Frail | 2.63 (2.49-2.78) | <0.001 |  | 2.78 (2.63-2.94) | <0.001 |  | 2.24 (2.12-2.38) | <0.001 |  | 1.91 (1.80-2.03) | <0.001 |
| P for trend |  | <0.001 |  |  | <0.001 |  |  | <0.001 |  |  | <0.001 |
| Frailty Index |  |  |  |  |  |  |  |  |  |  |  |
| Per 0.1 score increase | 1.62 (1.59-1.65) | <0.001 |  | 1.69 (1.66-1.72) | <0.001 |  | 1.59 (1.56-1.62) | <0.001 |  | 1.47 (1.45-1.50) | <0.001 |
| Non-frail | 1.00 (Ref) |  |  | 1.00 (Ref) |  |  | 1.00 (Ref) |  |  | 1.00 (Ref) |  |
| Prefrail | 1.64 (1.59-1.68) | <0.001 |  | 1.68 (1.63-1.73) | <0.001 |  | 1.58 (1.53-1.62) | <0.001 |  | 1.46 (1.42-1.50) | <0.001 |
| Frail | 2.88 (2.76-3.00) | <0.001 |  | 3.20 (3.06-3.34) | <0.001 |  | 2.74 (2.62-2.87) | <0.001 |  | 2.32 (2.22-2.42) | <0.001 |
| P for trend |  | <0.001 |  |  | <0.001 |  |  | <0.001 |  |  | <0.001 |
| **Stroke** |  |  |  |  |  |  |  |  |  |  |  |
| Physical Frailty |  |  |  |  |  |  |  |  |  |  |  |
| Continuous (per 1 score) | 1.25 (1.21-1.28) | <0.001 |  | 1.25 (1.21-1.28) | <0.001 |  | 1.20 (1.16-1.23) | <0.001 |  | 1.15 (1.12-1.19) | <0.001 |
| Non-frail | 1.00 (Ref) |  |  | 1.00 (Ref) |  |  | 1.00 (Ref) |  |  | 1.00 (Ref) |  |
| Prefrail | 1.28 (1.22-1.34) | <0.001 |  | 1.28 (1.22-1.35) | <0.001 |  | 1.22 (1.16-1.29) | <0.001 |  | 1.18 (1.12-1.24) | <0.001 |
| Frail | 2.10 (1.88-2.34) | <0.001 |  | 2.09 (1.88-2.34) | <0.001 |  | 1.80 (1.61-2.02) | <0.001 |  | 1.61 (1.43-1.80) | <0.001 |
| P for trend |  | <0.001 |  |  | <0.001 |  |  | <0.001 |  |  | <0.001 |
| Frailty Index |  |  |  |  |  |  |  |  |  |  |  |
| Per 0.1 score increase | 1.33 (1.29-1.37) | <0.001 |  | 1.32 (1.28-1.36) | <0.001 |  | 1.25 (1.21-1.29) | <0.001 |  | 1.17 (1.14-1.21) | <0.001 |
| Non-frail | 1.00 (Ref) |  |  | 1.00 (Ref) |  |  | 1.00 (Ref) |  |  | 1.00 (Ref) |  |
| Prefrail | 1.30 (1.24-1.36) | <0.001 |  | 1.26 (1.20-1.33) | <0.001 |  | 1.20 (1.14-1.26) | <0.001 |  | 1.13 (1.07-1.19) | <0.001 |
| Frail | 1.83 (1.68-1.99) | <0.001 |  | 1.86 (1.71-2.03) | <0.001 |  | 1.64 (1.50-1.79) | <0.001 |  | 1.44 (1.31-1.57) | <0.001 |
| P for trend |  | <0.001 |  |  | <0.001 |  |  | <0.001 |  |  | <0.001 |
| **Heart Failure** |  |  |  |  |  |  |  |  |  |  |  |
| Physical Frailty |  |  |  |  |  |  |  |  |  |  |  |
| Continuous (per 1 score) | 1.54 (1.51-1.57) | <0.001 |  | 1.56 (1.52-1.59) | <0.001 |  | 1.43 (1.40-1.46) | <0.001 |  | 1.35 (1.32-1.38) | <0.001 |
| Non-frail | 1.00 (Ref) |  |  | 1.00 (Ref) |  |  | 1.00 (Ref) |  |  | 1.00 (Ref) |  |
| Prefrail | 1.68 (1.61-1.76) | <0.001 |  | 1.70 (1.63-1.78) | <0.001 |  | 1.54 (1.47-1.61) | <0.001 |  | 1.43 (1.37-1.50) | <0.001 |
| Frail | 4.08 (3.76-4.43) | <0.001 |  | 4.20 (3.87-4.56) | <0.001 |  | 3.10 (2.85-3.38) | <0.001 |  | 2.55 (2.34-2.78) | <0.001 |
| P for trend |  | <0.001 |  |  | <0.001 |  |  | <0.001 |  |  | <0.001 |
| Frailty Index |  |  |  |  |  |  |  |  |  |  |  |
| Continuous (per 0.1 unit) | 1.76 (1.72-1.81) | <0.001 |  | 1.80 (1.76-1.85) | <0.001 |  | 1.63 (1.59-1.68) | <0.001 |  | 1.48 (1.44-1.52) | <0.001 |
| Non-frail | 1.00 (Ref) |  |  | 1.00 (Ref) |  |  | 1.00 (Ref) |  |  | 1.00 (Ref) |  |
| Prefrail | 1.80 (1.72-1.89) | <0.001 |  | 1.78 (1.70-1.87) | <0.001 |  | 1.63 (1.55-1.71) | <0.001 |  | 1.47 (1.40-1.54) | <0.001 |
| Frail | 3.45 (3.22-3.69) | <0.001 |  | 3.66 (3.42-3.92) | <0.001 |  | 2.91 (2.71-3.12) | <0.001 |  | 2.35 (2.19-2.53) | <0.001 |
| P for trend |  | <0.001 |  |  | <0.001 |  |  | <0.001 |  |  | <0.001 |
| **Atrial Fibrillation** |  |  |  |  |  |  |  |  |  |  |  |
| Physical Frailty |  |  |  |  |  |  |  |  |  |  |  |
| Continuous (per 1 score) | 1.22 (1.20-1.24) | <0.001 |  | 1.25 (1.23-1.27) | <0.001 |  | 1.21 (1.19-1.23) | <0.001 |  | 1.17 (1.15-1.19) | <0.001 |
| Non-frail | 1.00 (Ref) |  |  | 1.00 (Ref) |  |  | 1.00 (Ref) |  |  | 1.00 (Ref) |  |
| Prefrail | 1.24 (1.20-1.27) | <0.001 |  | 1.27 (1.23-1.31) | <0.001 |  | 1.22 (1.19-1.26) | <0.001 |  | 1.18 (1.14-1.22) | <0.001 |
| Frail | 2.05 (1.92-2.20) | <0.001 |  | 2.19 (2.05-2.34) | <0.001 |  | 1.96 (1.83-2.11) | <0.001 |  | 1.76 (1.63-1.89) | <0.001 |
| P for trend |  | <0.001 |  |  | <0.001 |  |  | <0.001 |  |  | <0.001 |
| Frailty Index |  |  |  |  |  |  |  |  |  |  |  |
| Continuous (per 0.1 unit) | 1.40 (1.37-1.42) | <0.001 |  | 1.42 (1.39-1.45) | <0.001 |  | 1.38 (1.35-1.40) | <0.001 |  | 1.30 (1.28-1.33) | <0.001 |
| Non-frail | 1.00 (Ref) |  |  | 1.00 (Ref) |  |  | 1.00 (Ref) |  |  | 1.00 (Ref) |  |
| Prefrail | 1.41 (1.37-1.46) | <0.001 |  | 1.40 (1.36-1.44) | <0.001 |  | 1.35 (1.31-1.39) | <0.001 |  | 1.28 (1.24-1.32) | <0.001 |
| Frail | 1.94 (1.84-2.05) | <0.001 |  | 2.09 (1.98-2.20) | <0.001 |  | 1.92 (1.82-2.03) | <0.001 |  | 1.71 (1.62-1.81) | <0.001 |
| P for trend |  | <0.001 |  |  | <0.001 |  |  | <0.001 |  |  | <0.001 |
| **Peripheral Artery Disease** |  |  |  |  |  |  |  |  |  |  |  |
| Physical Frailty |  |  |  |  |  |  |  |  |  |  |  |
| Continuous (per 1 score) | 1.35 (1.32-1.38) | <0.001 |  | 1.37 (1.34-1.40) | <0.001 |  | 1.27 (1.24-1.30) | <0.001 |  | 1.23 (1.20-1.26) | <0.001 |
| Non-frail | 1.00 (Ref) |  |  | 1.00 (Ref) |  |  | 1.00 (Ref) |  |  | 1.00 (Ref) |  |
| Prefrail | 1.39 (1.34-1.45) | <0.001 |  | 1.42 (1.36-1.48) | <0.001 |  | 1.31 (1.25-1.36) | <0.001 |  | 1.25 (1.20-1.30) | <0.001 |
| Frail | 2.72 (2.50-2.95) | <0.001 |  | 2.83 (2.60-3.08) | <0.001 |  | 2.21 (2.02-2.41) | <0.001 |  | 1.96 (1.79-2.14) | <0.001 |
| P for trend |  | <0.001 |  |  | <0.001 |  |  | <0.001 |  |  | <0.001 |
| Frailty Index |  |  |  |  |  |  |  |  |  |  |  |
| Continuous (per 0.1 unit) | 1.62 (1.58-1.66) | <0.001 |  | 1.65 (1.61-1.70) | <0.001 |  | 1.52 (1.48-1.56) | <0.001 |  | 1.44 (1.40-1.48) | <0.001 |
| Non-frail | 1.00 (Ref) |  |  | 1.00 (Ref) |  |  | 1.00 (Ref) |  |  | 1.00 (Ref) |  |
| Prefrail | 1.66 (1.59-1.73) | <0.001 |  | 1.65 (1.58-1.72) | <0.001 |  | 1.51 (1.45-1.58) | <0.001 |  | 1.43 (1.37-1.49) | <0.001 |
| Frail | 2.76 (2.58-2.94) | <0.001 |  | 2.90 (2.72-3.10) | <0.001 |  | 2.37 (2.21-2.54) | <0.001 |  | 2.10 (1.96-2.26) | <0.001 |
| P for trend |  | <0.001 |  |  | <0.001 |  |  | <0.001 |  |  | <0.001 |

Model 1 was adjusted age, sex, ethnicity. Model 2 was adjusted age, sex, ethnicity, education level, BMI, Townsend deprivation index, smoking status, alcohol consumption, and physical activity. Model 3 was further adjusted for hypertension and diabetes. Abbreviation: CI, Confidence Interval; HR, hazard ratio.

**Supplementary Table S15.** E-value analysis for quantifying the potential impact of unmeasured confounding on primary associations

| **Outcome** | **HR (95% CI)** | ***p* value** | **Point estimate E-value** | **Boundary E-value** |
| --- | --- | --- | --- | --- |
| **Cardiovascular Diseas**e |  |  |  |  |
| Physical Frailty (per 1 unit) | 1.21 (1.20-1.22) | <0.001 | 1.71 | 1.68 |
| Frailty Index (per 0.1 unit) | 1.40 (1.38-1.42) | <0.001 | 2.15 | 2.11 |
| **Coronary Heart Disease** |  |  |  |  |
| Physical Frailty (per 1 unit) | 1.23 (1.21-1.24) | <0.001 | 1.75 | 1.71 |
| Frailty Index (per 0.1 unit) | 1.49 (1.47-1.51) | <0.001 | 2.34 | 2.29 |
| **stroke** |  |  |  |  |
| Physical Frailty (per 1 unit) | 1.16 (1.13-1.19) | <0.001 | 1.59 | 1.51 |
| Frailty Index (per 0.1 unit) | 1.18 (1.14-1.22) | <0.001 | 1.65 | 1.55 |
| **Heart Failure** |  |  |  |  |
| Physical Frailty (per 1 unit) | 1.35 (1.32-1.38) | <0.001 | 2.04 | 1.98 |
| Frailty Index (per 0.1 unit) | 1.48 (1.44-1.52) | <0.001 | 2.33 | 2.24 |
| **Atrial Fibrillation** |  |  |  |  |
| Physical Frailty (per 1 unit) | 1.18 (1.16-1.20) | <0.001 | 1.63 | 1.58 |
| Frailty Index (per 0.1 unit) | 1.31 (1.29-1.34) | <0.001 | 1.96 | 1.9 |

Abbreviations: CI, confidence interval; HR, hazard ratio

**Supplementary Table S16.** Associations of physical frailty and frailty index with incident cardiovascular disease stratified by CKM stage.

| **CKM Stage** | **Events** | **Outcome** | **Crude** | | **Model 1** | | **Model 2** | |
| --- | --- | --- | --- | --- | --- | --- | --- | --- |
|  |  |  | **HR (95% CI)** | ***p*-value** | **HR (95% CI)** | ***p*-value** | **HR (95% CI)** | ***p*-value** |
| **Stage 0** |  | **Cardiovascular Disease** |  |  |  |  |  |  |
|  | 747 | Physical Frailty |  |  |  |  |  |  |
|  |  | Per 1 score increase | 1.34 (1.22-1.47) | <0.001 | 1.34 (1.22-1.47) | <0.001 | 1.30 (1.18-1.43) | <0.001 |
|  | 472 | Non-frail | 1.00 (Ref) |  | 1.00 (Ref) |  | 1.00 (Ref) |  |
|  | 251 | Prefrail | 1.31 (1.12-1.52) | <0.001 | 1.31 (1.13-1.53) | <0.001 | 1.26 (1.08-1.48) | 0.004 |
|  | 23 | Frail | 3.40 (2.23-5.16) | <0.001 | 3.41 (2.24-5.20) | <0.001 | 3.15 (2.04-4.86) | <0.001 |
|  |  | P for trend |  | <0.001 |  | <0.001 |  | <0.001 |
|  | 747 | Frailty Index |  |  |  |  |  |  |
|  |  | Per 0.1 score increase | 1.50 (1.35-1.67) | <0.001 | 1.49 (1.34-1.66) | <0.001 | 1.45 (1.30-1.62) | <0.001 |
|  | 460 | Non-frail | 1.00 (Ref) |  | 1.00 (Ref) |  | 1.00 (Ref) |  |
|  | 252 | Prefrail | 1.46 (1.26-1.71) | <0.001 | 1.48 (1.27-1.73) | <0.001 | 1.45 (1.24-1.69) | <0.001 |
|  | 35 | Frail | 2.60 (1.84-3.66) | <0.001 | 2.45 (1.74-3.46) | <0.001 | 2.25 (1.58-3.21) | <0.001 |
|  |  | P for trend |  | <0.001 |  | <0.001 |  | <0.001 |
| **Stage 1** |  | **Cardiovascular Disease** |  |  |  |  |  |  |
|  | 1700 | Physical Frailty |  |  |  |  |  |  |
|  |  | Per 1 score increase | 1.17 (1.10-1.24) | <0.001 | 1.20 (1.13-1.28) | <0.001 | 1.15 (1.08-1.23) | <0.001 |
|  | 1028 | Non-frail | 1.00 (Ref) |  | 1.00 (Ref) |  | 1.00 (Ref) |  |
|  | 611 | Prefrail | 1.08 (0.97-1.19) | 0.147 | 1.12 (1.01-1.23) | 0.031 | 1.07 (0.96-1.18) | 0.208 |
|  | 56 | Frail | 2.47 (1.89-3.23) | <0.001 | 2.72 (2.08-3.57) | <0.001 | 2.32 (1.76-3.07) | <0.001 |
|  |  | P for trend |  | <0.001 |  | <0.001 |  | <0.001 |
|  | 1700 | Frailty Index |  |  |  |  |  |  |
|  |  | Per 0.1 score increase | 1.45 (1.35-1.56) | <0.001 | 1.51 (1.41-1.62) | <0.001 | 1.45 (1.35-1.56) | <0.001 |
|  | 991 | Non-frail | 1.00 (Ref) |  | 1.00 (Ref) |  | 1.00 (Ref) |  |
|  | 627 | Prefrail | 1.48 (1.34-1.64) | <0.001 | 1.54 (1.40-1.71) | <0.001 | 1.48 (1.34-1.64) | <0.001 |
|  | 82 | Frail | 2.00 (1.59-2.50) | <0.001 | 2.19 (1.75-2.74) | <0.001 | 1.99 (1.59-2.51) | <0.001 |
|  |  | P for trend |  | <0.001 |  | <0.001 |  | <0.001 |
| **Stage 2** |  | **Cardiovascular Disease** |  |  |  |  |  |  |
|  | 24264 | Physical Frailty |  |  |  |  |  |  |
|  |  | Per 1 score increase | 1.32 (1.31-1.34) | <0.001 | 1.32 (1.30-1.34) | <0.001 | 1.27 (1.25-1.29) | <0.001 |
|  | 11994 | Non-frail | 1.00 (Ref) |  | 1.00 (Ref) |  | 1.00 (Ref) |  |
|  | 10566 | Prefrail | 1.38 (1.34-1.42) | <0.001 | 1.38 (1.34-1.42) | <0.001 | 1.31 (1.28-1.35) | <0.001 |
|  | 1502 | Frail | 2.54 (2.41-2.68) | <0.001 | 2.54 (2.41-2.68) | <0.001 | 2.18 (2.06-2.31) | <0.001 |
|  |  | P for trend |  | <0.001 |  | <0.001 |  | <0.001 |
|  | 24264 | Frailty Index |  |  |  |  |  |  |
|  |  | Per 0.1 score increase | 1.56 (1.54-1.59) | <0.001 | 1.56 (1.54-1.59) | <0.001 | 1.49 (1.46-1.51) | <0.001 |
|  | 10836 | Non-frail | 1.00 (Ref) |  | 1.00 (Ref) |  | 1.00 (Ref) |  |
|  | 10607 | Prefrail | 1.56 (1.52-1.60) | <0.001 | 1.54 (1.50-1.58) | <0.001 | 1.46 (1.42-1.50) | <0.001 |
|  | 2821 | Frail | 2.62 (2.52-2.73) | <0.001 | 2.62 (2.51-2.73) | <0.001 | 2.32 (2.22-2.42) | <0.001 |
|  |  | P for trend |  | <0.001 |  | <0.001 |  | <0.001 |
| **Stage 3** |  | **Cardiovascular Disease** |  |  |  |  |  |  |
|  | 25706 | Physical Frailty |  |  |  |  |  |  |
|  |  | Per 1 score increase | 1.22 (1.20-1.24) | <0.001 | 1.27 (1.25-1.28) | <0.001 | 1.21 (1.19-1.23) | <0.001 |
|  | 13546 | Non-frail | 1.00 (Ref) |  | 1.00 (Ref) |  | 1.00 (Ref) |  |
|  | 10660 | Prefrail | 1.24 (1.21-1.27) | <0.001 | 1.30 (1.26-1.33) | <0.001 | 1.23 (1.20-1.26) | <0.001 |
|  | 1291 | Frail | 2.02 (1.91-2.14) | <0.001 | 2.26 (2.13-2.39) | <0.001 | 1.93 (1.82-2.05) | <0.001 |
|  |  | P for trend |  | <0.001 |  | <0.001 |  | <0.001 |
|  | 25706 | Frailty Index |  |  |  |  |  |  |
|  |  | Per 0.1 score increase | 1.41 (1.38-1.43) | <0.001 | 1.48 (1.46-1.51) | <0.001 | 1.41 (1.39-1.43) | <0.001 |
|  | 12884 | Non-frail | 1.00 (Ref) |  | 1.00 (Ref) |  | 1.00 (Ref) |  |
|  | 10507 | Prefrail | 1.41 (1.37-1.45) | <0.001 | 1.44 (1.40-1.47) | <0.001 | 1.37 (1.33-1.40) | <0.001 |
|  | 2315 | Frail | 2.06 (1.97-2.15) | <0.001 | 2.40 (2.30-2.51) | <0.001 | 2.14 (2.04-2.24) | <0.001 |
|  |  | P for trend |  | <0.001 |  | <0.001 |  | <0.001 |

Model 1 was adjusted for age, sex, and ethnicity. Model 2 was further adjusted for education level, Townsend deprivation index, smoking status, alcohol consumption, physical activity, and body mass index. Diabetes and hypertension were not included in the CKM stage-stratified models because they are components of CKM staging and may overlap with frailty index construction, potentially leading to over-adjustment.

**Supplementary Table S17.** Sensitivity analyses using a modified frailty index excluding cardiometabolic and cardiovascular-related deficits for the association with incident cardiovascular outcomes.

| **Outcome** | **Crude** | | **Model 1** | | **Model 2** | |
| --- | --- | --- | --- | --- | --- | --- |
|  | **HR (95% CI)** | ***p*-value** | **HR (95% CI)** | ***p*-value** | **HR (95% CI)** | ***p*-value** |
| **Cardiovascular Disease** |  |  |  |  |  |  |
| Frailty Index |  |  |  |  |  |  |
| Per 0.1 score increase | 1.36 (1.35-1.37) | <0.001 | 1.42 (1.41-1.43) | <0.001 | 1.35 (1.34-1.37) | <0.001 |
| Non-frail | 1.00 (Ref) |  | 1.00 (Ref) |  | 1.00 (Ref) |  |
| Prefrail | 1.36 (1.34-1.39) | <0.001 | 1.40 (1.37-1.42) | <0.001 | 1.33 (1.31-1.36) | <0.001 |
| Frail | 2.00 (1.95-2.06) | <0.001 | 2.21 (2.15-2.27) | <0.001 | 1.96 (1.91-2.01) | <0.001 |
| P for trend |  | <0.001 |  | <0.001 |  | <0.001 |
| **Coronary Heart Disease** |  |  |  |  |  |  |
| Frailty Index |  |  |  |  |  |  |
| Per 0.1 score increase | 1.44 (1.42-1.46) | <0.001 | 1.52 (1.50-1.54) | <0.001 | 1.43 (1.41-1.45) | <0.001 |
| Non-frail | 1.00 (Ref) |  | 1.00 (Ref) |  | 1.00 (Ref) |  |
| Prefrail | 1.45 (1.41-1.49) | <0.001 | 1.51 (1.47-1.55) | <0.001 | 1.42 (1.39-1.46) | <0.001 |
| Frail | 2.28 (2.20-2.37) | <0.001 | 2.57 (2.48-2.67) | <0.001 | 2.23 (2.15-2.32) | <0.001 |
| P for trend |  | <0.001 |  | <0.001 |  | <0.001 |
| **Stroke** |  |  |  |  |  |  |
| Frailty Index |  |  |  |  |  |  |
| Per 0.1 score increase | 1.20 (1.17-1.24) | <0.001 | 1.22 (1.19-1.26) | <0.001 | 1.16 (1.13-1.20) | <0.001 |
| Non-frail | 1.00 (Ref) |  | 1.00 (Ref) |  | 1.00 (Ref) |  |
| Prefrail | 1.18 (1.12-1.24) | <0.001 | 1.18 (1.12-1.23) | <0.001 | 1.12 (1.07-1.18) | <0.001 |
| Frail | 1.48 (1.37-1.59) | <0.001 | 1.55 (1.44-1.67) | <0.001 | 1.38 (1.28-1.49) | <0.001 |
| P for trend |  | <0.001 |  | <0.001 |  | <0.001 |
| **Heart Failure** |  |  |  |  |  |  |
| Frailty Index |  |  |  |  |  |  |
| Per 0.1 score increase | 1.50 (1.47-1.54) | <0.001 | 1.56 (1.53-1.60) | <0.001 | 1.43 (1.40-1.47) | <0.001 |
| Non-frail | 1.00 (Ref) |  | 1.00 (Ref) |  | 1.00 (Ref) |  |
| Prefrail | 1.55 (1.48-1.62) | <0.001 | 1.57 (1.50-1.64) | <0.001 | 1.44 (1.38-1.51) | <0.001 |
| Frail | 2.52 (2.37-2.68) | <0.001 | 2.76 (2.59-2.93) | <0.001 | 2.24 (2.10-2.39) | <0.001 |
| P for trend |  | <0.001 |  | <0.001 |  | <0.001 |
| **Atrial Fibrillation** |  |  |  |  |  |  |
| Frailty Index |  |  |  |  |  |  |
| Per 0.1 score increase | 1.25 (1.23-1.27) | <0.001 | 1.29 (1.27-1.32) | <0.001 | 1.26 (1.24-1.28) | <0.001 |
| Non-frail | 1.00 (Ref) |  | 1.00 (Ref) |  | 1.00 (Ref) |  |
| Prefrail | 1.26 (1.23-1.30) | <0.001 | 1.28 (1.24-1.32) | <0.001 | 1.25 (1.21-1.29) | <0.001 |
| Frail | 1.62 (1.55-1.70) | <0.001 | 1.79 (1.71-1.87) | <0.001 | 1.68 (1.60-1.76) | <0.001 |
| P for trend |  | <0.001 |  | <0.001 |  | <0.001 |
| **Peripheral Artery Disease** |  |  |  |  |  |  |
| Frailty Index |  |  |  |  |  |  |
| Per 0.1 score increase | 1.44 (1.41-1.47) | <0.001 | 1.48 (1.45-1.52) | <0.001 | 1.37 (1.34-1.40) | <0.001 |
| Non-frail | 1.00 (Ref) |  | 1.00 (Ref) |  | 1.00 (Ref) |  |
| Prefrail | 1.48 (1.42-1.54) | <0.001 | 1.50 (1.44-1.56) | <0.001 | 1.39 (1.33-1.45) | <0.001 |
| Frail | 2.26 (2.14-2.39) | <0.001 | 2.42 (2.29-2.56) | <0.001 | 2.01 (1.90-2.13) | <0.001 |
| P for trend |  | <0.001 |  | <0.001 |  | <0.001 |

Model 1 was adjusted age, sex, ethnicity. Model 2 was adjusted age, sex, ethnicity, education level, BMI, Townsend deprivation index, smoking status, alcohol consumption, and physical activity. Abbreviation: CI, Confidence Interval; HR, hazard ratio.

**Supplementary Table S18.** Comparison of baseline characteristics between the complete-case and multiple-imputation analyses

| Variable | Type | Level | Complete-case analysis (N = 330,197) | Multiple-imputation analysis (N = 444,774) | SMD |
| --- | --- | --- | --- | --- | --- |
| Sex | Categorical | Female | 182,167 (55.2%) | 248,610 (55.9%) | 0.015 |
| Sex | Categorical | Male | 148,030 (44.8%) | 196,164 (44.1%) | 0.015 |
| Age, years | Continuous |  | 56.08 (8.09) | 56.04 (8.09) | 0.004 |
| Ethnicity | Categorical | White | 311,416 (94.3%) | 418,921 (94.2%) | 0.005 |
| Ethnicity | Categorical | Black | 1,974 (0.6%) | 2,660 (0.6%) | 0 |
| Ethnicity | Categorical | Other | 12,305 (3.7%) | 17,081 (3.8%) | 0.006 |
| Ethnicity | Categorical | Asian | 4,502 (1.4%) | 6,112 (1.4%) | 0.001 |
| Body mass index, kg/m2 | Continuous |  | 27.23 (4.66) | 27.25 (4.71) | 0.006 |
| Townsend deprivation index | Continuous |  | -1.39 (3.02) | -1.36 (3.06) | 0.009 |
| Alcohol consumption | Categorical | < 3x/w | 159,462 (48.3%) | 215,777 (48.6%) | 0.005 |
| Alcohol consumption | Categorical | >= 3x/w | 146,023 (44.2%) | 195,153 (43.9%) | 0.006 |
| Alcohol consumption | Categorical | Never | 24,712 (7.5%) | 33,499 (7.5%) | 0.002 |
| Education | Categorical | High | 109,673 (33.2%) | 149,169 (33.5%) | 0.007 |
| Education | Categorical | Intermediate | 130,274 (39.5%) | 173,985 (39.1%) | 0.007 |
| Education | Categorical | Low | 90,250 (27.3%) | 121,620 (27.3%) | 0 |
| Smoking status | Categorical | Current | 34,034 (10.3%) | 46,040 (10.4%) | 0.001 |
| Smoking status | Categorical | Never | 183,842 (55.9%) | 248,448 (56.1%) | 0.003 |
| Smoking status | Categorical | Previous | 111,087 (33.8%) | 148,766 (33.6%) | 0.004 |
| CKM stage | Categorical | Stage 0 | 15,814 (4.8%) | 21,048 (4.7%) | 0.003 |
| CKM stage | Categorical | Stage 1 | 31,005 (9.4%) | 42,186 (9.5%) | 0.003 |
| CKM stage | Categorical | Stage 2 | 161,414 (48.9%) | 219,342 (49.3%) | 0.009 |
| CKM stage | Categorical | Stage 3 | 121,964 (36.9%) | 162,198 (36.5%) | 0.01 |
| Incident CVD | Categorical | 0 | 277,780 (84.1%) | 374,607 (84.2%) | 0.003 |
| Incident CVD | Categorical | 1 | 52,417 (15.9%) | 70,167 (15.8%) | 0.003 |
| Physical frailty score | Continuous |  | 0.56 (0.80) | 0.56 (0.81) | 0.006 |
| Physical frailty category | Categorical | Frail | 9,819 (3.0%) | 13,750 (3.1%) | 0.006 |
| Physical frailty category | Categorical | Non-frail | 194,567 (59.2%) | 262,771 (59.1%) | 0.003 |
| Physical frailty category | Categorical | Prefrail | 124,084 (37.8%) | 168,253 (37.8%) | 0.001 |
| Frailty index | Continuous |  | 0.12 (0.07) | 0.12 (0.07) | 0.001 |
| Frailty index category | Categorical | Frail | 18,540 (5.6%) | 25,200 (5.7%) | 0.002 |
| Frailty index category | Categorical | Non-frail | 194,969 (59.0%) | 262,137 (58.9%) | 0.002 |
| Frailty index category | Categorical | Prefrail | 116,688 (35.3%) | 157,437 (35.4%) | 0.001 |

Data are presented as mean (standard deviation) for continuous variables and n (%) for categorical variables. For the multiple-imputation analysis, means, standard deviations, counts, and percentages were summarized across 20 imputed datasets; counts represent the average number of participants across the completed datasets and were rounded to the nearest integer. Standardized mean differences were used to compare the distributions of baseline characteristics between the complete-case and multiple-imputation analyses. An absolute SMD <0.10 was considered to indicate a negligible difference. CKM, cardiovascular-kidney-metabolic; CVD, cardiovascular disease; SMD, standardized mean difference.

**Supplementary Table 19.** Comparison of the associations of physical frailty and frailty index with incident CVD between complete-case and multiple-imputation analyses.

| **Outcome** | **Complete-case analysis crude** | | **Multiple-imputation analysis crude** | | **Complete-case analysis Model 1** | | **Multiple-imputation analysis Model 1** | | **Complete-case analysis Model 2** | | **Multiple-imputation analysis Model 2** | |
| --- | --- | --- | --- | --- | --- | --- | --- | --- | --- | --- | --- | --- |
|  | **HR (95% CI)** | ***p*-value** | **HR (95% CI)** | ***p*-value** | **HR (95% CI)** | ***p*-value** | **HR (95% CI)** | ***p*-value** | **HR (95% CI)** | ***p*-value** | **HR (95% CI)** | ***p*-value** |
| **Cardiovascular Disease** |  |  |  |  |  |  |  |  |  |  |  |  |
| Physical Frailty |  |  |  |  |  |  |  |  |  |  |  |  |
| Per 1 score increase | 1.30 (1.28–1.31) | <0.001 | 1.30 (1.29–1.31) | <0.001 | 1.32 (1.31–1.33) | <0.001 | 1.33 (1.31–1.34) | <0.001 | 1.25 (1.24-1.27) | <0.001 | 1.21 (1.20–1.22) | <0.001 |
| Non-frail | Reference |  | Reference |  | Reference |  | Reference |  | 1.00 (Ref) |  | Reference |  |
| Prefrail | 1.33 (1.31–1.35) | <0.001 | 1.34 (1.32–1.37) | <0.001 | 1.36 (1.34–1.38) | <0.001 | 1.38 (1.36–1.40) | <0.001 | 1.28 (1.26-1.31) | <0.001 | 1.24 (1.22–1.26) | <0.001 |
| Frail | 2.44 (2.34–2.53) | <0.001 | 2.44 (2.36–2.52) | <0.001 | 2.56 (2.46–2.66) | <0.001 | 2.58 (2.49–2.66) | <0.001 | 2.15 (2.06-2.24) | <0.001 | 1.89 (1.82–1.96) | <0.001 |
| P for trend |  | <0.001 |  | <0.001 |  | <0.001 |  | <0.001 |  | <0.001 |  | <0.001 |
| Frailty Index |  |  |  |  |  |  |  |  |  |  |  |  |
| Per 0.1 score increase | 1.53 (1.51–1.54) | <0.001 | 1.53 (1.52–1.55) | <0.001 | 1.57 (1.55–1.59) | <0.001 | 1.58 (1.56–1.59) | <0.001 | 1.48 (1.47-1.50) | <0.001 | 1.44 (1.42–1.45) | <0.001 |
| Non-frail | Reference |  | Reference |  | Reference |  | Reference |  | 1.00 (Ref) |  | Reference |  |
| Prefrail | 1.52 (1.50–1.55) | <0.001 | 1.53 (1.51–1.55) | <0.001 | 1.54 (1.51–1.56) | <0.001 | 1.54 (1.52–1.57) | <0.001 | 1.45 (1.43-1.48) | <0.001 | 1.41 (1.38–1.43) | <0.001 |
| Frail | 2.48 (2.40–2.55) | <0.001 | 2.48 (2.42–2.55) | <0.001 | 2.67 (2.60–2.76) | <0.001 | 2.68 (2.61–2.75) | <0.001 | 2.34 (2.27-2.41) | <0.001 | 2.13 (2.08–2.19) | <0.001 |
| P for trend |  | <0.001 |  | <0.001 |  | <0.001 |  | <0.001 |  | <0.001 |  | <0.001 |

Values are presented as hazard ratios with 95% confidence intervals. Model 1 was adjusted for age, sex, and ethnicity. Model 2 was additionally adjusted for education, Townsend deprivation index, smoking status, alcohol consumption, physical activity, and body mass index. Estimates from 20 imputed datasets were combined according to Rubin’s rules. HR, hazard ratio; CI, confidence interval; CVD, cardiovascular disease.

**Supplementary Table S20.** Associations of physical frailty and frailty index with incident CVD using Fine–Gray competing-risk models.

| **Outcome** | **Crude** | | **Model 1** | | **Model 2** | |
| --- | --- | --- | --- | --- | --- | --- |
|  | **sHR (95% CI)** | ***p*-value** | **sHR (95% CI)** | ***p*-value** | **sHR (95% CI)** | ***p*-value** |
| **Cardiovascular Disease** |  |  |  |  |  |  |
| Physical Frailty |  |  |  |  |  |  |
| Per 1 score increase | 1.283 (1.271–1.296) | <0.001 | 1.300 (1.287–1.312) | <0.001 | 1.194 (1.181–1.206) | <0.001 |
| Non-frail | Reference |  | Reference |  | Reference |  |
| Prefrail | 1.318 (1.295–1.342) | <0.001 | 1.343 (1.320–1.368) | <0.001 | 1.210 (1.188–1.232) | <0.001 |
| Frail | 2.350 (2.261–2.443) | <0.001 | 2.429 (2.334–2.527) | <0.001 | 1.818 (1.744–1.896) | <0.001 |
| P for trend |  | <0.001 |  | <0.001 |  | <0.001 |
| Frailty Index |  |  |  |  |  |  |
| Per 0.1 score increase | 1.513 (1.497–1.529) | <0.001 | 1.549 (1.532–1.567) | <0.001 | 1.416 (1.399–1.433) | <0.001 |
| Non-frail | Reference |  | Reference |  | Reference |  |
| Prefrail | 1.513 (1.486–1.541) | <0.001 | 1.520 (1.492–1.548) | <0.001 | 1.391 (1.365–1.417) | <0.001 |
| Frail | 2.426 (2.355–2.500) | <0.001 | 2.596 (2.518–2.677) | <0.001 | 2.094 (2.028–2.162) | <0.001 |
| P for trend |  | <0.001 |  | <0.001 |  | <0.001 |

Values are presented as subdistribution hazard ratios with 95% confidence intervals. Death occurring before incident cardiovascular disease was treated as the competing event. The crude model was unadjusted. Model 1 was adjusted for age, sex, and ethnicity. Model 2 was additionally adjusted for educational level, Townsend deprivation index, body mass index, smoking status, alcohol consumption, and physical activity. Abbreviations: CI, confidence interval; CVD, cardiovascular disease; sHR, subdistribution hazard ratio.

**Supplementary Figure S1.** Dose–response associations of physical frailty with CVD outcomes. Restricted cubic spline curves from Cox proportional hazards models show associations of frailty index with CVD outcomes. Solid lines represent hazard ratios and shaded areas indicate 95% CIs. *P* values for overall and nonlinearity are shown. Adjusted for variables in Model 3. **A** Cardiovascular Disease; **B** Coronary Heart Disease; **C** Stroke; **D** Heart Failure; **E** Atrial Fibrillation; **F** Peripheral Artery Disease.

**Supplementary Figure S2**. Cumulative incidence curves for cardiovascular disease outcomes stratified by physical frailty. Frailty patients were associated with increased cumulative incidence across all outcomes (Gray’s test p < 0.001). A Cardiovascular Disease; B Coronary Heart Disease; C Stroke; D Heart Failure; E Atrial Fibrillation; F Peripheral Artery Disease.

**Supplementary Figure S3.** Subgroup analyses of frailty status and incident CHD. HRs and 95% CIs for incident CVD across frailty status are shown for subgroups defined by age, sex, smoking status, alcohol consumption, hypertension, and diabetes. Abbreviations: CHD, Coronary Heart Disease; HR, hazard ratio; CI, confidence interval.

**Supplementary Figure S4.** Subgroup analyses of frailty status and incident heart failure. HRs and 95% CIs for incident heart failure across frailty status are shown for subgroups defined by age, sex, smoking status, alcohol consumption, hypertension, and diabetes. Abbreviations: HR, hazard ratio; CI, confidence interval.

**Supplementary Figure S5.** Subgroup analyses of frailty status and incident atrial fibrillation. HRs and 95% CIs for incident atrial fibrillation across frailty status are shown for subgroups defined by age, sex, smoking status, alcohol consumption, hypertension, and diabetes. Abbreviations: HR, hazard ratio; CI, confidence interval.
